# Ultrafast anisotropic dynamics of hyperbolic nanolight pulse propagation

**DOI:** 10.1126/sciadv.adi4407

**Published:** 2023-08-25

**Authors:** Xin Zhang, Qizhi Yan, Weiliang Ma, Tianning Zhang, Xiaosheng Yang, Xinliang Zhang, Peining Li

**Affiliations:** ^1^ Wuhan National Laboratory for Optoelectronics and School of Optical and Electronic Information, Huazhong University of Science and Technology, Wuhan 430074, China.; ^2^ Optics Valley Laboratory, Hubei 430074, China.; ^3^ Xidian University, Xi’an 710126, China.

## Abstract

Polariton pulses—transient light-matter hybrid excitations—traveling through anisotropic media can lead to unusual optical phenomena in space and time. However, studying these pulses presents challenges with their anisotropic, ultrafast, and nanoscale field variations. Here, we demonstrate the creation, observation, and control of polariton pulses, with in-plane hyperbolic dispersion, on anisotropic crystal surfaces by using a time-resolved nanoimaging technique and our developed high-dimensional data processing. We capture and analyze movies of distinctive pulse spatiotemporal dynamics, including curved ultraslow energy flow trajectories, anisotropic dissipation, and dynamical misalignment between phase and group velocities. Our approach enables analysis of polariton pulses in the wave vector time domain, demonstrating a time-domain polaritonic topological transition from lenticular to hyperbolic dispersion contours and the ability to study the polariton-induced time-varying optical forces. Our findings promise to facilitate the study of diverse space-time phenomena at extreme scales and drive advances in ultrafast nanoimaging.

## Teaser

Ultrafast nano-optical movies of hyperbolic nanolight pulses on anisotropic crystal surfaces are captured and analyzed.

## INTRODUCTION

The study of light pulse propagation through various materials has led to unexpected discoveries in optics, uncovering phenomena such as superluminal and subluminal behavior (*1*, *2*), negative phase and group velocities (*3*–*6*), and nonlinear optical effects (*7*). Materials with extreme anisotropy (*8*–*14*), characterized by opposing permittivity tensor elements, offer promising opportunities to produce highly anisotropic nanolight pulses with hyperbolic dispersion (*6*, *15*, *16*). These nanolight pulses, called hyperbolic polariton (HP) pulses, arise from the interaction between transient electromagnetic radiations and material excitations, such as collective electrons or lattice vibrations (*17*–*19*). They exhibit nonintuitive spatiotemporal features, including a misalignment between phase velocities **v**_p_ and group velocities **v**_g_ (*6*–*20*). In extremely confined HPs, **v**_p_ is orthogonal to **v**_g_, which contrasts with conventional isotropic pulses that have **v**_p_ parallel to **v**_g_ [antiparallel for backward waves (*3*)]. These intriguing properties provide exciting opportunities for ultrafast and directional control of nanophotonic energies (*12*, *21*, *22*), with potential applications in negative refraction (*23*, *24*), anomalous focusing (*25*–*27*), molecular sensing (*28*, *29*), and hyperlensing (*30*, *31*). However, studying the spatiotemporal dynamics of HP pulse propagation is challenging due to its transient, anisotropic, and subwavelength-scale nature.

Here, we demonstrate the experimental visualization of the in-plane anisotropic propagation of HP pulses on calcite surfaces, using a phase-sensitive time-resolved infrared nanoimaging technique. We select calcite crystals for their ability to exhibit highly anisotropic HP propagation patterns at sizes ranging up to tens of micrometers (*12*). Scattering-type scanning near-field optical microscopy (s-SNOM) provides high spatial resolution reaching down to ~10 nm in broad spectral ranges from visible to terahertz frequencies (*32*, *33*), benefiting the material characterization and nanophotonic structure diagnosis (*34*). Our technique combines time-domain interferometry and s-SNOM, providing unparalleled imaging resolution for measuring the complex-valued HP pulse fields. To address the challenges associated with the high-dimensional space-time [i.e., two-dimensional (2D) space and time] imaging nature of our approach, including time cost, instability, and background noise, we develop a comprehensive strategy encompassing data acquisition, processing, and interpretation. Our nanoimaging approach and high-dimensional data processing enable the simultaneous assessment of anisotropic velocities **v**_p_ and **v**_g_ and dynamic visualization of their vectorial misalignment, providing unprecedented insights into the anisotropic spatiotemporal dynamics of HP pulses.

## RESULTS

**Fig. 1.**
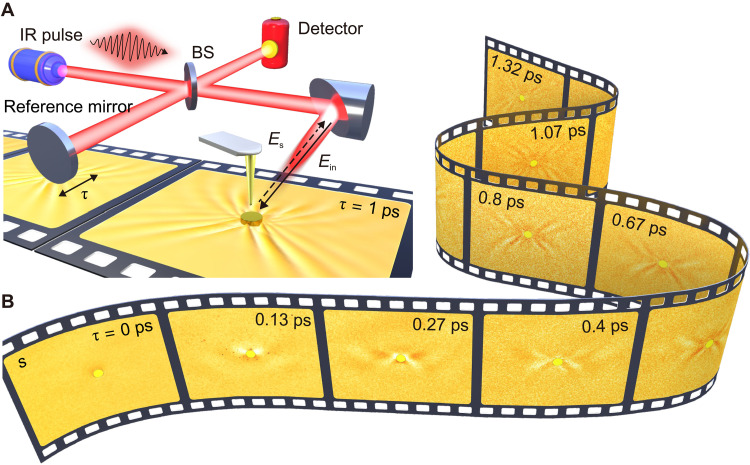
Time-domain interferometry nanoimaging of HP pulse propagation. (**A**) Experimental setup. IR, infrared; BS, beam splitter. Note that the incident laser pulses are perpendicular to the propagating direction of polaritons (see the illustration) for suppressing the asymmetry of polariton fields excited on either side of the gold disk. (**B**) Experimental near-field snapshots (amplitude signal *s*, raw data). The entire raw datasets are up to 80 frames (fig. S1).

The experimental setup used in this study is illustrated in Fig. 1A. Mid-infrared pulses of 100-fs duration are directed into an asymmetric Michelson interferometer, with the pulse spectral range covering the Reststrahlen band of calcite (see Materials and Methods). The object beam illuminates a gold disk that is fabricated on the (100) surface of a calcite crystal, acting as an optical antenna and converting free-space light pulses to highly anisotropic HP pulses that propagate along the surface. A metallic SNOM tip is then brought close to the sample surface to scatter the excited HP pulse fields, which are superimposed with background illumination. The tip-scattered fields are cross-correlated with reference pulses and recorded by an infrared detector, while the interferometer time delay τ is adjusted by moving the reference mirror. To address instability caused by collecting the complete interferogram for each tip position on the sample surface, we develop a more robust scanning strategy where the tip is scanned over the surface at each fixed time delay τ, resulting in a 2D near-field snapshot of the HP pulse fields with nanoscale spatial resolution (see Materials and Methods). These snapshots are repeated when varying the time delay τ, creating a nano-movie—a high-dimensional space-time imaging dataset—that displays the anisotropic in-plane propagation of HP pulses, including their electric-field variations, anisotropic energy flow, and dissipation.

Figure 1B showcases experimental near-field amplitude snapshots that vividly capture bright and dark butterfly-like fringes of HP pulses emitted from the gold disk. These raw transient data (fig. S1) illustrate the dynamic evolution of the fringes in space and time, from the initial emergence through the build-up to the eventual annihilation. To gain a complete understanding of the evolution, a video comprising 317 frames, reconstructed via data interpolation (note S1), is available in movie S1. At short time delays (τ < 0.4 ps), a single fringe emerges near the disk, and with τ increasing, HP pulses propagate away from the disk, creating multiple fringes of larger concave curvatures. The fringes disappear at longer time delays (τ > 1.3 ps), indicating their relatively long lifetime. The validity and high quality of our spatiotemporal experiments are further confirmed by time-domain simulations that reproduce all observed HP pulse features (see fig. S2).

**Fig. 2.**
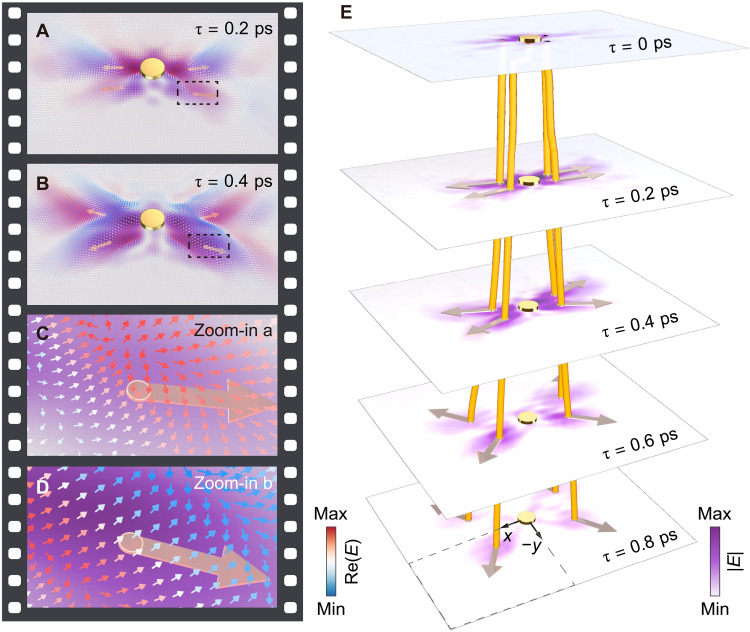
Space-time mapping of HP pulse velocity vectors. (**A** and **B**) Two background-subtracted experimental near-field snapshots showing the velocity pseudo-vector textures of HP pulses (see the complete movie shown in movie S2). Small colored arrows show local distributions of fringe velocity **v**_f_, with the colors showing the real part of the near fields of HPs. The large pale gold arrows indicate the centroids of four HP beams, respectively. Their directions are aligned with the corresponding centroid velocity **v**_c_. (**C** and **D**) Zoom-in images taken from the dashed boxes in (A) and (B), respectively. (**E**) Time-dependent background-subtracted experimental near-field amplitude snapshots revealing the space-time evolution of butterfly-shaped polariton beams (the complete movie provided in movie S3), giving rise to centroid trajectories of each beam (yellow lines). Arrows indicate the directions of **v**_c_ in each moment.

To accurately quantify the propagation of HP pulses, we use high-dimensional Fourier transform (FT) filtering to subtract the background fields and the field components of frequencies outside the Reststrahlen band (fig. S5 and detailed descriptions in note S2). The resulting filtered near-field data (real part) exhibit HP pulse fringes with intrinsic phase variations, which we captured in a sequence of snapshots shown in movie S2 and in Fig. 2 (A and B). We determine the fringe velocity **v**_f_ by analyzing the gradient variations in the time-domain image sequence (see Materials and Methods), with **v**_f_ parallel to the phase velocity **v**_p_ and the momentum **k**. In Fig. 2 (A and B), we plot the time-dependent local distributions of **v**_f_ as arrows (see zoom-ins in Fig. 2, C and D), each aligned with its corresponding **v**_f_. At short time delays (Fig. 2C), we observe diverse orientations of **v**_f_ due to the appearance of small-**k** HPs. However, as time progressed, the arrows point uniformly in the same direction (Fig. 2D), indicating the appearance and dominance of highly directional large-**k** HPs in pulse-field distribution.

Figure 2E provides background-subtracted near-field amplitude snapshots (movie S3) that illustrate the anisotropic energy flow of HP pulses. This image sequence features butterfly-shaped polariton beams with four nearly symmetric branches, providing a clear visualization of how HP energies are emitted from the disk (τ = 0 ps), spread out along the sample surface (τ ≥ 0.2 ps), and dissipate over time. To analyze these dynamic processes, we track the centroid trajectory of each beam branch in the high-dimensional space-time (see Materials and Methods), represented by four yellow lines in Fig. 2E. From these trajectories, we extract the centroid velocities **v**_c_ (indicated by pale gold arrows in Fig. 2), which correspond to the averaged **v**_g_ that gauges the overall energy flow (see Materials and Methods). We find that **v**_c_ is not parallel to **v**_f_ (i.e., **v**_g_ being not parallel to **v**_p_; see Fig. 2, C and D), confirming the most prominent feature of HPs—the vectorial misalignment between the velocities **v**_p_ and **v**_g_.

**Fig. 3.**
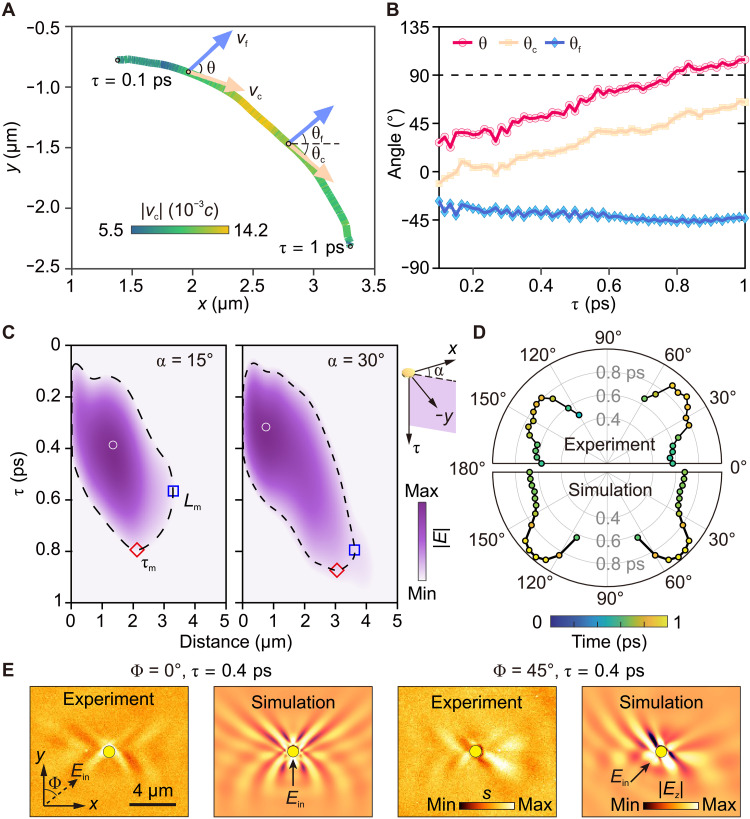
Anisotropic spatiotemporal dynamics and incidence-dependent control of HP pulse propagation. (**A**) Centroid trajectory of the HP pulse beam (marked by a dashed box in Fig. 2E). The color gradient of the trajectory curve indicates the magnitude of the centroid velocity **v**_c_ that is obtained from time intervals. Blue and yellow arrows indicate the direction of the fringe velocity **v**_f_ and the centroid velocity **v**_c_ obtained at the centroid, respectively. Their orientation angles θ_c_ and θ_f_ are defined with respect to the *x* axis. θ is the angle between **v**_c_ and **v**_f_. (**B**) Time-dependent variations of θ_c_, θ_f_, and θ during the HP pulse propagation. (**C**) Space-time evolution slices of near-field amplitudes of the HP pulse beam for two different propagating directions (the angle α defined according to the inset). Black dashed lines show the profiles where the pulse amplitudes decay to 1/e of the maximum of the whole beam. Maximum decay lengths *L*_m_ and maximum decay times τ_m_ are marked by blue squares and red rhombs, respectively. White circles indicate the maximum amplitude in each slice. (**D**) Experimental anisotropic decay time of HP pulses (top), showing good agreement with numerical simulation (bottom). The slight difference between the left and right in the experimental result is attributed to the imperfect alignment of the laser pulses with respect to the *y* direction. (**E**) Near-field distributions of HP pulses launched in different excitation directions (illustrated by arrows). Left: Experimental near-field amplitude snapshots. Right: Simulated near-field amplitude snapshots.

In Fig. 3A, we show in detail the trajectory of a specific centroid in the *x*-*y* space, revealing a curved path that goes against the ray-like trajectories usually observed in monochromatic imaging experiments (*12*). The observed pulse beams and their curved centroid paths can be demonstrated by our time-domain simulations, which can be explained by the coherent superposition of different frequency components of HP pulses (fig. S8). We further analyze this anomalous curved energy flowing by examining the centroid velocity **v**_c_. The orientation angle θ_c_ of **v**_c_ continuously increases with time during the pulse propagation (Fig. 3B), while the fringe velocity **v**_f_ exhibits a nearly constant angle (|θ_f_| ⁓ 45°) taken from positions of the centroid. By comparing the θ_f_ value with the theoretical HP dispersion, we infer that it corresponds to the frequency component ω = 1470 cm^−1^ (the center of the Reststrahlen band) of the pulse fields. The misalignment angle θ between **v**_c_ and **v**_f_ increases with time, up to more than 90° after τ ⁓ 0.8 ps, which corroborates the extreme HP anisotropy. By examining the time-varying magnitude of **v**_c_ (the color gradient in Fig. 3A), we demonstrate the ultraslow energy flow of HP pulses with the velocity |**v**_c_| as slow as 0.005*c* (*c* being the speed of light in free space). Moreover, we unveil the dynamic phenomena of the energy-flowing acceleration (τ < 0.6 ps) and deceleration (τ > 0.6 ps), which can be explained by the frequency dispersion of HP pulses. The observations of the curved energy-flowing trajectories, along with the acceleration and deceleration, unambiguously demonstrate the potential of our nanoimaging approach in investigating the peculiar spatiotemporal physics of anisotropic polaritons.

Figure 3C delves into the anisotropic dissipations of HP pulses by showing two cross-section slices from the high-dimensional dataset (the one depicted in Fig. 2E). These slices reveal the time-varying near-field amplitudes of a single HP pulse beam in two azimuthal directions (15° and 30°, with respect to the *x* axis). In both directions, we observe the pulse packet (shown in purple) moving away from the disk (located at the coordinate origin) with increasing time. The maximum amplitudes (marked by white circles) do not appear at the disk, but rather at a distance, indicating the energy accumulation during propagation. To quantify the anisotropic decay, we extract the 1/e value of the maximum of the overall beam in the high-dimensional space-time (dashed lines in Fig. 3C; see also fig. S9) and derive the maximum values of the profiles along the position axis and the time axis as the maximum decay lengths *L*_m_ and maximum decay times τ_m_ (see symbols marked in Fig. 3C), respectively. It is remarkable that *L*_m_ and τ_m_ do not coincide (see symbols marked in Fig. 3C), indicating the large group velocity dispersion of HP pulses.

Figure 3D displays the extracted τ_m_ for various directions (top panel), which promptly reveals the in-plane anisotropic decay of HP pulses, in good agreement with numerical simulations (bottom panel; see note S3 for details). Note that we did not consider the 2D spreading decay of disk-excited polaritons (*6*, *8*, *9*). Therefore, the experimentally obtained quantities are smaller than the intrinsic values. These spatiotemporal observations directly unveil the extreme space-time anisotropy and the strong group velocity dispersion of HP pulses, which hold potential applications in anisotropic waveguiding (*21*, *22*), nanoscale heat transfer (*35*), and peculiar light-matter interactions (*36*).

In Fig. 3E, we demonstrate the capability of regulating the anisotropic propagation of HP pulses by altering their excitation direction with respect to the optic axis (OA) of the calcite crystal. We present two experimental near-field amplitude snapshots, acquired at different incident angles relative to the crystal's OA (i.e., the *y* direction). Our observations show that when HP pulses are excited parallel to the *y* axis, they exhibit propagation fringe patterns with mirror symmetry relative to the *y* direction. Conversely, varying the incidence angle to 45° results in asymmetry fringe patterns of HP pulses. Our simulation results validate the effectiveness of controlling the propagation symmetry and dynamics of HP pulses, showing excellent agreement with the experimental data. This regulating effect is attributed to the disk antenna acting as an in-plane dipole emitter that is able to selectively excite the momentum components of HPs [see details in (*37*)]. These findings offer opportunities for shaping the propagation behavior of HP pulses, which could have implications for applications in topological photonics (*38*), ultrafast information transfer, and processing (*19*).

**Fig. 4.**
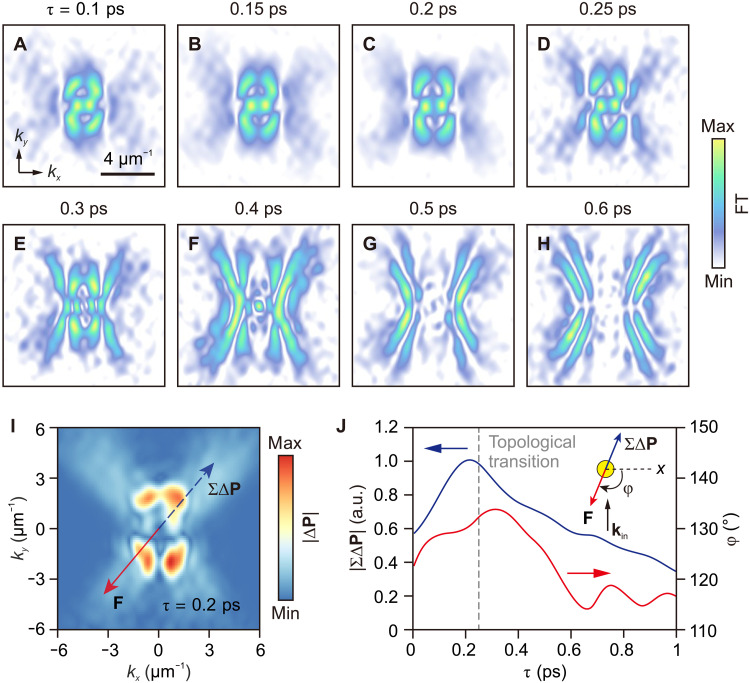
Time-domain topological transition and time-varying optical pulling forces. (**A** to **H**) Time-dependent wave vector distribution of HP pulses revealing the IFCs from lenticular to hyperbolic. The time-domain topological transition occurs at around τ = 0.25 ps when the hyperbolic IFCs gradually dominate. Note that the antenna excites the polariton pulses composed of broad frequency components, resulting in the simultaneous presence of both hyperbolic and lenticular polariton components. (**I**) Momentum variation at τ = 0.2 ps. The blue dashed arrow shows the orientation of summing momentum variation and the red arrow indicates the direction of the corresponding optical force. (**J**) Summing momentum variation (blue line) and the orientation of optical forces (red line, the angle ϕ defined according to the inset) as a function of time. The gray dashed line marks the time when the topological transition occurs. a.u. arbitrary units.

The spatiotemporal characteristics of polariton pulses are determined by their extremely anisotropic wave vectors **k**. In our experiments, we are able to monitor polariton wave vectors in the (**k**, *t*) domain, allowing us to demonstrate a time-dependent topological transition for isofrequency contours (IFCs) in **k**-space. We achieve this by using the 2D FT to convert near-field data into domains of in-plane momenta (*k*_x_ and *k*_y_) and time delay τ (as detailed in note S4, Fig. 4, A to H, and movie S4). In this analysis, we include the data with laser frequencies outside the Reststrahlen band. The processed dataset reveals a lenticular-shaped IFC at short time delays (τ < 0.25 ps), which undergoes a topological transition at around τ = 0.25 ps. During this transition, the lenticular IFC flattens out, and the hyperbolic IFC emerges at larger *k* values. Subsequently, the lenticular IFC disappears, and the remaining hyperbolic IFCs exhibit a decreasing opening angle with increasing time. We also analyze the polariton field confinement by obtaining the largest wave vector of the IFC as a function of τ, as shown in fig. S12. We show that this time-domain topological transition can be tuned by adjusting the initial phases of the illumination pulses, as discussed in fig. S13. Topological transitions arising from the modifications of the polariton dispersion in anisotropic materials enable the enhancement of the local density of photonic states for light-matter interactions, typically reported by static experiments (*12*, *27*). Our results represent the time-domain topological transition, which holds potential for various applications on ultrafast time scales, such as sensing, waveguiding, and imaging.

After characterizing the spatiotemporal dynamics of HP pulses and observing the time-domain topological transition, we demonstrate the ability to explore the optical pulling forces of HP pulses. Recent theoretical studies predict that variations in the wave vector **k**, specifically the transition topological of their IFCs, can induce optical pulling forces when light scatters off an object within a designed photonic environment (*39*). By directly accessing the time-varying wave vectors and light momentum **P** (with **P** = ℏ**k**, where ℏ is the reduced Planck constant), we are able to experimentally evaluate the optical forces of polariton pulses induced on the disk antenna (note S5). In Fig. 4I, we show the distribution of the time-dependent variations of light momentum |∆**P**| at τ = 0.2 ps. We observe that the patterns of |∆**P**| consist of two parts, with the low-**k** lenticular parts experiencing a larger increment than the high-**k** hyperbolic ones. The patterns of |∆**P**| are asymmetric, which we attribute to the oblique incidence and imperfect alignment of the laser pulses. We estimate the resulting force induced on the disk by summing the changes in momentum for all wave vector components using the equation of

$$F(\tau )=-\frac{\sum \text{Δ}P}{\text{Δ}\tau }=-\frac{\iint |\text{Δ}N|\hbar kdk_{x}dk_{y}}{\text{Δ}\tau }$$ (1)

where ∆*N* is the change for each wave vector component **k** (see note S5 for details). The direction of the force **F** is opposite to that of the momentum of the incident light (illustrated in Fig. 4J), representing a pulling force. Figure 4J shows that the value of |∑∆**P**| (blue line, indicating the magnitude of **F**) and the direction angle of the force **F** (red line) vary over time. Both values show maximum at time delays close to the topological transition (indicated by a dashed gray line), which is in line with the theoretical prediction that the topological transition can enhance the optical forces (*39*). The value of |∑∆**P**| reaches a maximum at around τ = 0.21 ps and subsequently decreases. The angle ϕ of the **F** direction shows its maximum at around τ = 0.31 ps. Taking into account the parameters of the pulse laser used in the experiment and assuming an efficiency of 1% for the antenna converting free-space pulses to polariton pulses (*40*), we estimate the maximum transient force **F** to be on the order of approximately 100 pN (see note S5 for details).

## DISCUSSION

In summary, we have demonstrated the spatiotemporal characteristics of extremely anisotropic nanolight pulses using the time-domain interferometric nanoimaging technique. In combination with diverse polarized light excitations, such as circularly polarized light (*41*, *42*), we envision that our approach can be applied to reveal various exotic polariton dynamics. It also holds potential for multiphysics space-time analysis, as demonstrated by the evaluation of momentum topology–induced optical pulling forces exerted by polariton pulses on an object situated on a naturally occurring hyperbolic surface. The time-dependent evolution of polariton wave vectors and associated topological transitions observed in our study offers exciting opportunities for dynamically tailoring the local density of photonic states in various applications (*19*) and exploring high-dimensional time-varying optics (*43*, *44*). We anticipate that the use of artificial intelligence techniques (*45*) and machine learning algorithms (*46*) will become increasingly important to accelerate the acquisition of high-dimensional spatiotemporal data and improve the imaging quality and accuracy. The spatiotemporal dynamics of HP pulses demonstrated here are essential for developing anisotropy-driven ultrafast nanophotonic and thermal applications.

## MATERIALS AND METHODS

### Materials and sample fabrications

We used 1 cm–by–1 cm–sized calcite substrates with characteristic (100) plane, which were commercially available by mechanical cleavage from bulk calcite single crystals. Gold disk antennas were fabricated on the calcite surface via standard electron beam lithography. The disk patterns were written on the resist (polymethyl methacrylate: 495/A4, thickness ≈ 100 nm) spin-coated on the calcite substrate. A conductive polymer (AR-PC 5090) was spin-coated on the resist to prevent charge accumulation because of the weak conductivity of calcite. The standard lift-off procedure was carried out after the e-beam evaporation of Ti (3 nm)/Au (60 nm) onto the developed resist.

### Time-domain interferometric measurements

For time-domain interferometric nanoimaging experiments, we used a commercial nano-FTIR system from Neaspec GmbH based on an atomic force microscope (AFM). The Pt-coated AFM tip oscillates vertically with an amplitude of about 70 nm at a frequency Ω ≈ 250 kHz (Arrow NCPt, NanoWorld). It is illuminated by a broadband laser pulse of 100-fs duration (range from 1000 to 2000 cm^−1^; see fig. S3). A piezo-controlled linearly moving mirror is used to precisely control the time delay τ between the tip-scattered field and the reference field. The 2D mappings were carried out at different time delays with a temporal resolution of Δτ = 16.7 fs (corresponding to the reference-mirror moving interval of 2.5 μm) satisfying the band-pass sampling (see note S1 for details). The interferometric detector signal was demodulated at a higher harmonic *n*Ω (*n* ≥ 2), yielding near-field amplitude *s*_n_ and phase ϕ_n_ images as a function of both the 2D (*x*, *y*) coordinates and time delay τ. In this work, we recorded the signal of the second order for producing a raw space-time imaging dataset (see Fig. 1B and fig. S1), e.g., the real part of near fields, *E*(*x*,*y*,τ) *= s*_2_ cos(ϕ_2_). We display the mappings of *s*_1_ in Fig. 3E since the higher-order signal is weak for an incidence angle of 45°.

### Polariton velocity vectors reconstruction

We used the Horn–Schunck (HS) algorithm to extract the pulse fringe velocities **v**_f_. The HS algorithm is well-known for computing the optical flow from image sequences (*47*), which was recently used to analyze the plasmonic flow in the photoemission electron microscopy experiment (*48*). It estimates a velocity vector field (**v**_x_, **v**_y_) by minimizing the equation *E*_HS_ ≈ ∫∫(*I*_x_*v*_x_ + *I*_y_*v*_x_ + *I*_t_)^2^d*x*d*y*, where *I*_x,y_ and *I*_t_ are the spatial and temporal image intensity derivatives in each pixel, respectively. Specifically, we applied the HS algorithm to analyze the image sequences of the real part of filtered spatiotemporal data after normalizing them to the range of [0,1] for each frame, thus extracting the pixel-wise local distributions of time-dependent fringe velocities. In Fig. 3 (A and B), the fringe velocities **v**_f_ at the centroid location were obtained by averaging fringe velocities in the range of 0.5 μm by 0.5 μm to make the trend smoother.

Taking the antenna center as the coordinate origin, we divided the 2D real space into four quadrants and processed each quadrant (i.e., each polariton beam), respectively. At each time delay, we removed the data whose absolute value is less than the 1/e value of the maxima of this quadrant so that the processed polariton beam is more distinct and less noisy. Then, we calculated the centroid position by the equation $x_{c}=\frac{\sum I_{i}x_{i}}{\sum I_{i}}$ and $y_{c}=\frac{\sum I_{i}y_{i}}{\sum I_{i}}$, where *I*_i_ is the absolute value of processed near-field data in each pixel, *x*_i_ and *y*_i_ are the corresponding coordinates. We extracted the centroid velocities **v**_c_ by calculating the centroid movement between time steps, i.e., $v_{c}(\tau )=\frac{p(\tau )-p(\tau -d\tau )}{d\tau }$, where **p**(τ) is the position vector (*x*_c_(τ), *y*_c_(τ)) at each time delay τ.

### Permittivity of the calcite crystal used in simulations

The uniaxial permittivity (ɛ_⊥_ for the component in the direction perpendicular to the OA and ɛ_∥_ for parallel to the OA) of the calcite crystal was calculated by Lorentz oscillator models, where two Lorentz oscillators were used for ɛ_⊥_ and one for ɛ_∥_ according to Eqs. 2 and 3

$$\varepsilon_{⊥}=\varepsilon_{\infty ,1}\left(1+\frac{\omega_{\mathrm{LO},1}^{2}-\omega_{\mathrm{TO},1}^{2}}{\omega_{\mathrm{TO},1}^{2}-\omega^{2}-i\omega {\text{Γ}}_{1}}+\frac{\omega_{\mathrm{LO},2}^{2}-\omega_{\mathrm{TO},2}^{2}}{\omega_{\mathrm{TO},2}^{2}-\omega^{2}-i\omega {\text{Γ}}_{2}}\right)$$ (2)

$$\varepsilon_{∥}=\varepsilon_{\infty ,3}\left(1+\frac{\omega_{\mathrm{LO},3}^{2}-\omega_{\mathrm{TO},3}^{2}}{\omega_{\mathrm{TO},3}^{2}-\omega^{2}-i\omega {\text{Γ}}_{3}}\right)$$ (3)

where ω_TO_ and ω_LO_ are transverse optical (TO) and longitudinal optical (LO) phonon frequencies, respectively. Γ is the damping constant. ɛ_∞_ is the high-frequency permittivity. For ɛ_⊥_, we took ɛ_∞,1_= 2.7; ω_TO,1_ = 712 cm^−1^; ω_LO,1_ = 715 cm^−1^; Γ_1_= 5 cm^−1^; ω_TO,2_ = 1410 cm^−1^; ω_LO,2_ = 1550 cm^−1^; Γ_2_= 10 cm^−1^. For ɛ_∥_, we took ɛ_∞,3_= 2.4; ω_TO,3_ = 871 cm^−1^; ω_LO,3_ = 890 cm^−1^; Γ_3_= 3 cm^−1^. The parameters above are from (*12*).

### Numerical simulations

We used a finite-difference time-domain simulation software (Lumerical FDTD) to simulate the dynamics of near-field distributions (*E*_z_) of polariton pulses propagating along the calcite’s surface.

To validate the measured space-time features of HP pulses, we simulated disk-launched polariton distributions when a gold disk (height, 50 nm; diameter, 1 μm) was placed on top of a calcite substrate (with an angle of 23.3° between the OA and the surface). A p-polarized broadband plane-wave pulse was directed obliquely onto the sample, illuminating a gold disk and launching HP pulses. The incident angle is 30° relative to the surface, and the OA of calcite is in the plane of incidence. The spectrum of the broadband pulse is Gaussian-shaped and central at 1500 cm^−1^, approximating the infrared pulse used in the experiment. The horizontal simulation range covers 50 μm by 50 μm, and Bloch boundary conditions are applied. Note that the simulation range is sufficiently large to avoid interference that could arise from the boundary. We recorded the time-varying near-field distributions (*E*_z_) at 100 nm above the surface. Simulated results shown in Fig. 3E were obtained by changing the illuminating orientation.

To study the decay time of HPs in different lateral directions shown in Fig. 3D, we monitored the time-dependent near-field distributions at 50 nm above the calcite substrate with a dipole (polarized perpendicular to the surface) exciting at 500 nm above the sample. Perfectly matched layers are used as boundary conditions.

## Supplementary Materials

### This PDF file includes:

Notes S1 to S5 Figs. S1 to S13 Legends for movies S1 to S4 References

## References

[1] R. W. Boyd, D. J. Gauthier, Controlling the velocity of light pulses. Science 326, 1074–1077 (2009). 19965419 10.1126/science.1170885

[2] K. L. Tsakmakidis, O. Hess, R. W. Boyd, X. Zhang, Ultraslow waves on the nanoscale. Science 358, eaan5196 (2017). 29051348 10.1126/science.aan5196

[3] H. Gersen, T. J. Karle, R. J. Engelen, W. Bogaerts, J. P. Korterik, N. F. van Hulst, T. F. Krauss, L. Kuipers, Direct observation of Bloch harmonics and negative phase velocity in photonic crystal waveguides. Phys. Rev. Lett. 94, 123901 (2005). 15903920 10.1103/PhysRevLett.94.123901

[4] G. M. Gehring, A. Schweinsberg, C. Barsi, N. Kostinski, R. W. Boyd, Observation of backward pulse propagation through a medium with a negative group velocity. Science 312, 895–897 (2006). 16690861 10.1126/science.1124524

[5] G. Dolling, C. Enkrich, M. Wegener, C. M. Soukoulis, S. Linden, Simultaneous negative phase and group velocity of light in a metamaterial. Science 312, 892–894 (2006). 16690860 10.1126/science.1126021

[6] E. Yoxall, M. Schnell, A. Y. Nikitin, O. Txoperena, A. Woessner, M. B. Lundeberg, F. Casanova, L. E. Hueso, F. H. L. Koppens, R. Hillenbrand, Direct observation of ultraslow hyperbolic polariton propagation with negative phase velocity. Nat. Photonics 9, 674–678 (2015).

[7] R. W. Boyd, *Nonlinear Optics* (Academic Press, 2020).

[8] S. Dai, Z. Fei, Q. Ma, A. S. Rodin, M. Wagner, A. S. McLeod, M. K. Liu, W. Gannett, W. Regan, K. Watanabe, T. Taniguchi, M. Thiemens, G. Dominguez, A. H. Castro Neto, A. Zettl, F. Keilmann, P. Jarillo-Herrero, M. M. Fogler, D. N. Basov, Tunable phonon polaritons in atomically thin van der Waals crystals of boron nitride. Science 343, 1125–1129 (2014). 24604197 10.1126/science.1246833

[9] P. Li, I. Dolado, F. J. Alfaro-Mozaz, F. Casanova, L. E. Hueso, S. Liu, J. H. Edgar, A. Y. Nikitin, S. Velez, R. Hillenbrand, Infrared hyperbolic metasurface based on nanostructured van der Waals materials. Science 359, 892–896 (2018). 29472478 10.1126/science.aaq1704

[10] W. Ma, P. Alonso-Gonzalez, S. Li, A. Y. Nikitin, J. Yuan, J. Martin-Sanchez, J. Taboada-Gutierrez, I. Amenabar, P. Li, S. Velez, C. Tollan, Z. Dai, Y. Zhang, S. Sriram, K. Kalantar-Zadeh, S. T. Lee, R. Hillenbrand, Q. Bao, In-plane anisotropic and ultra-low-loss polaritons in a natural van der Waals crystal. Nature 562, 557–562 (2018). 30356185 10.1038/s41586-018-0618-9

[11] Z. Zheng, N. Xu, S. L. Oscurato, M. Tamagnone, F. Sun, Y. Jiang, Y. Ke, J. Chen, W. Huang, W. L. Wilson, A. Ambrosio, S. Deng, H. Chen, A mid-infrared biaxial hyperbolic van der Waals crystal. Sci. Adv. 5, eaav8690 (2019). 31139747 10.1126/sciadv.aav8690 PMC6534390

[12] W. Ma, G. Hu, D. Hu, R. Chen, T. Sun, X. Zhang, Q. Dai, Y. Zeng, A. Alù, C. W. Qiu, P. Li, Ghost hyperbolic surface polaritons in bulk anisotropic crystals. Nature 596, 362–366 (2021). 34408329 10.1038/s41586-021-03755-1

[13] N. C. Passler, X. Ni, G. Hu, J. R. Matson, G. Carini, M. Wolf, M. Schubert, A. Alù, J. D. Caldwell, T. G. Folland, A. Paarmann, Hyperbolic shear polaritons in low-symmetry crystals. Nature 602, 595–600 (2022). 35197618 10.1038/s41586-021-04328-y PMC8866127

[14] G. Hu, W. Ma, D. Hu, J. Wu, C. Zheng, K. Liu, X. Zhang, X. Ni, J. Chen, X. Zhang, Q. Dai, J. D. Caldwell, A. Paarmann, A. Alù, P. Li, C. W. Qiu, Real-space nanoimaging of hyperbolic shear polaritons in a monoclinic crystal. Nat. Nanotechnol. 18, 64–70 (2023). 36509927 10.1038/s41565-022-01264-4

[15] Y. Kurman, R. Dahan, H. H. Sheinfux, K. Wang, M. Yannai, Y. Adiv, O. Reinhardt, L. H. G. Tizei, S. Y. Woo, J. Li, J. H. Edgar, M. Kociak, F. H. L. Koppens, I. Kaminer, Spatiotemporal imaging of 2D polariton wave packet dynamics using free electrons. Science 372, 1181–1186 (2021). 34112689 10.1126/science.abg9015

[16] A. J. Sternbach, S. H. Chae, S. Latini, A. A. Rikhter, Y. Shao, B. Li, D. Rhodes, B. Kim, P. J. Schuck, X. Xu, X. Y. Zhu, R. D. Averitt, J. Hone, M. M. Fogler, A. Rubio, D. N. Basov, Programmable hyperbolic polaritons in van der Waals semiconductors. Science 371, 617–620 (2021). 33542134 10.1126/science.abe9163

[17] D. N. Basov, M. M. Fogler, F. J. Garcia de Abajo, Polaritons in van der Waals materials. Science 354, aag1992 (2016). 27738142 10.1126/science.aag1992

[18] T. Low, A. Chaves, J. D. Caldwell, A. Kumar, N. X. Fang, P. Avouris, T. F. Heinz, F. Guinea, L. Martin-Moreno, F. Koppens, Polaritons in layered two-dimensional materials. Nat. Mater. 16, 182–194 (2017). 27893724 10.1038/nmat4792

[19] Q. Zhang, G. Hu, W. Ma, P. Li, A. Krasnok, R. Hillenbrand, A. Alù, C. W. Qiu, Interface nano-optics with van der Waals polaritons. Nature 597, 187–195 (2021). 34497390 10.1038/s41586-021-03581-5

[20] J. D. Caldwell, A. V. Kretinin, Y. Chen, V. Giannini, M. M. Fogler, Y. Francescato, C. T. Ellis, J. G. Tischler, C. R. Woods, A. J. Giles, M. Hong, K. Watanabe, T. Taniguchi, S. A. Maier, K. S. Novoselov, Sub-diffractional volume-confined polaritons in the natural hyperbolic material hexagonal boron nitride. Nat. Commun. 5, 5221 (2014). 25323633 10.1038/ncomms6221

[21] F. J. Rodriguez-Fortuno, G. Marino, P. Ginzburg, D. O'Connor, A. Martinez, G. A. Wurtz, A. V. Zayats, Near-field interference for the unidirectional excitation of electromagnetic guided modes. Science 340, 328–330 (2013). 23599487 10.1126/science.1233739

[22] J. Lin, J. P. Mueller, Q. Wang, G. Yuan, N. Antoniou, X. C. Yuan, F. Capasso, Polarization-controlled tunable directional coupling of surface plasmon polaritons. Science 340, 331–334 (2013). 23599488 10.1126/science.1233746

[23] A. J. Sternbach, S. L. Moore, A. Rikhter, S. Zhang, R. Jing, Y. Shao, B. S. Y. Kim, S. Xu, S. Liu, J. H. Edgar, A. Rubio, C. Dean, J. Hone, M. M. Fogler, D. N. Basov, Negative refraction in hyperbolic hetero-bicrystals. Science 379, 555–557 (2023). 36758086 10.1126/science.adf1065

[24] H. Hu, N. Chen, H. Teng, R. Yu, M. Xue, K. Chen, Y. Xiao, Y. Qu, D. Hu, J. Chen, Z. Sun, P. Li, F. J. G. de Abajo, Q. Dai, Gate-tunable negative refraction of mid-infrared polaritons. Science 379, 558–561 (2023). 36758071 10.1126/science.adf1251

[25] J. Duan, G. Alvarez-Perez, A. I. F. Tresguerres-Mata, J. Taboada-Gutierrez, K. V. Voronin, A. Bylinkin, B. Chang, S. Xiao, S. Liu, J. H. Edgar, J. I. Martin, V. S. Volkov, R. Hillenbrand, J. Martin-Sanchez, A. Y. Nikitin, P. Alonso-Gonzalez, Planar refraction and lensing of highly confined polaritons in anisotropic media. Nat. Commun. 12, 4325 (2021). 34267201 10.1038/s41467-021-24599-3 PMC8282686

[26] J. Martin-Sanchez, J. Duan, J. Taboada-Gutierrez, G. Alvarez-Perez, K. V. Voronin, I. Prieto, W. Ma, Q. Bao, V. S. Volkov, R. Hillenbrand, A. Y. Nikitin, P. Alonso-Gonzalez, Focusing of in-plane hyperbolic polaritons in van der Waals crystals with tailored infrared nanoantennas. Sci. Adv. 7, eabj0127 (2021). 34623915 10.1126/sciadv.abj0127 PMC8500510

[27] H. Hu, N. Chen, H. Teng, R. Yu, Y. Qu, J. Sun, M. Xue, D. Hu, B. Wu, C. Li, J. Chen, M. Liu, Z. Sun, Y. Liu, P. Li, S. Fan, F. J. Garcia de Abajo, Q. Dai, Doping-driven topological polaritons in graphene/α-MoO_3_ heterostructures. Nat. Nanotechnol. 17, 940–946 (2022). 35982316 10.1038/s41565-022-01185-2 PMC9477736

[28] M. Autore, P. Li, I. Dolado, F. J. Alfaro-Mozaz, R. Esteban, A. Atxabal, F. Casanova, L. E. Hueso, P. Alonso-Gonzalez, J. Aizpurua, A. Y. Nikitin, S. Velez, R. Hillenbrand, Boron nitride nanoresonators for phonon-enhanced molecular vibrational spectroscopy at the strong coupling limit. Light Sci. Appl. 7, 17172 (2018). 30839544 10.1038/lsa.2017.172 PMC6060053

[29] A. Bylinkin, M. Schnell, M. Autore, F. Calavalle, P. Li, J. Taboada-Gutierrez, S. Liu, J. H. Edgar, F. Casanova, L. E. Hueso, P. Alonso-Gonzalez, A. Y. Nikitin, R. Hillenbrand, Real-space observation of vibrational strong coupling between propagating phonon polaritons and organic molecules. Nat. Photonics 15, 197–202 (2021).

[30] S. Dai, Q. Ma, T. Andersen, A. S. McLeod, Z. Fei, M. K. Liu, M. Wagner, K. Watanabe, T. Taniguchi, M. Thiemens, F. Keilmann, P. Jarillo-Herrero, M. M. Fogler, D. N. Basov, Subdiffractional focusing and guiding of polaritonic rays in a natural hyperbolic material. Nat. Commun. 6, 6963 (2015). 25902364 10.1038/ncomms7963 PMC4421822

[31] P. Li, M. Lewin, A. V. Kretinin, J. D. Caldwell, K. S. Novoselov, T. Taniguchi, K. Watanabe, F. Gaussmann, T. Taubner, Hyperbolic phonon-polaritons in boron nitride for near-field optical imaging and focusing. Nat. Commun. 6, 7507 (2015). 26112474 10.1038/ncomms8507 PMC4491815

[32] F. Keilmann, R. Hillenbrand, Near-field microscopy by elastic light scattering from a tip. Philos. Trans. A. Math. Phys. Eng. Sci. 362, 787–805 (2004). 15306494 10.1098/rsta.2003.1347

[33] T. L. Cocker, V. Jelic, R. Hillenbrand, F. A. Hegmann, Nanoscale terahertz scanning probe microscopy. Nat. Photonics 15, 558–569 (2021).

[34] X. Chen, D. Hu, R. Mescall, G. You, D. N. Basov, Q. Dai, M. Liu, Modern scattering-type scanning near-field optical microscopy for advanced material research. Adv. Mater. 31, e1804774 (2019). 30932221 10.1002/adma.201804774

[35] K. J. Tielrooij, N. C. H. Hesp, A. Principi, M. B. Lundeberg, E. A. A. Pogna, L. Banszerus, Z. Mics, M. Massicotte, P. Schmidt, D. Davydovskaya, D. G. Purdie, I. Goykhman, G. Soavi, A. Lombardo, K. Watanabe, T. Taniguchi, M. Bonn, D. Turchinovich, C. Stampfer, A. C. Ferrari, G. Cerullo, M. Polini, F. H. L. Koppens, Out-of-plane heat transfer in van der Waals stacks through electron-hyperbolic phonon coupling. Nat. Nanotechnol. 13, 41–46 (2018). 29180742 10.1038/s41565-017-0008-8

[36] N. Rivera, I. Kaminer, Light-matter interactions with photonic quasiparticles. Nat. Rev. Phys. 2, 538–561 (2020).

[37] C. Hu, T. Sun, Y. Zeng, W. Ma, Z. Dai, X. Yang, X. Zhang, P. Li, Source-configured symmetry-broken hyperbolic polaritons. eLight 3, 14 (2023).

[38] T. Ozawa, H. M. Price, A. Amo, N. Goldman, M. Hafezi, L. Lu, M. C. Rechtsman, D. Schuster, J. Simon, O. Zilberberg, I. Carusotto, Topological photonics. Rev. Mod. Phys. 91, 015006 (2019).

[39] H. Li, Y. Cao, B. Shi, T. Zhu, Y. Geng, R. Feng, L. Wang, F. Sun, Y. Shi, M. A. Miri, M. Nieto-Vesperinas, C. W. Qiu, W. Ding, Momentum-topology-induced optical pulling force. Phys. Rev. Lett. 124, 143901 (2020). 32338962 10.1103/PhysRevLett.124.143901

[40] P. Pons-Valencia, F. J. Alfaro-Mozaz, M. M. Wiecha, V. Biolek, I. Dolado, S. Velez, P. Li, P. Alonso-Gonzalez, F. Casanova, L. E. Hueso, L. Martin-Moreno, R. Hillenbrand, A. Y. Nikitin, Launching of hyperbolic phonon-polaritons in h-BN slabs by resonant metal plasmonic antennas. Nat. Commun. 10, 3242 (2019). 31324759 10.1038/s41467-019-11143-7 PMC6642108

[41] L. Xiong, Y. Li, D. Halbertal, M. Sammon, Z. Sun, S. Liu, J. H. Edgar, T. Low, M. M. Fogler, C. R. Dean, A. J. Millis, D. N. Basov, Polaritonic vortices with a half-integer charge. Nano Lett. 21, 9256–9261 (2021). 34709832 10.1021/acs.nanolett.1c03175

[42] M. Wang, G. Hu, S. Chand, M. Cotrufo, Y. Abate, K. Watanabe, T. Taniguchi, G. Grosso, C. W. Qiu, A. Alù, Spin-orbit-locked hyperbolic polariton vortices carrying reconfigurable topological charges. eLight 2, 12 (2022).

[43] N. Engheta, Four-dimensional optics using time-varying metamaterials. Science 379, 1190–1191 (2023). 36952405 10.1126/science.adf1094

[44] E. Galiffi, R. Tirole, S. X. Yin, H. N. Li, S. Vezzoli, P. A. Huidobro, M. G. Silveirinha, R. Sapienza, A. Alù, J. B. Pendry, Photonics of time-varying media. Adv. Photonics 4, 014002 (2022).

[45] Y. H. Tang, J. C. Fan, X. W. Li, J. Z. Ma, M. H. Qi, C. X. Yu, W. L. Gao, Physics-informed recurrent neural network for time dynamics in optical resonances. Nat. Comput. Sci. 2, 169–178 (2022). 10.1038/s43588-022-00215-2 38177446

[46] X. Chen, S. Xu, S. Shabani, Y. Zhao, M. Fu, A. J. Millis, M. M. Fogler, A. N. Pasupathy, M. Liu, D. N. Basov, Machine learning for optical scanning probe nanoscopy. Adv. Mater., 2109171 (2022). 10.1002/adma.202109171 36333118

[47] B. K. P. Horn, B. G. Schunck, Determining optical flow. Artif. Intell. 17, 185–203 (1981).

[48] Y. Dai, Z. Zhou, A. Ghosh, R. S. K. Mong, A. Kubo, C. B. Huang, H. Petek, Plasmonic topological quasiparticle on the nanometre and femtosecond scales. Nature 588, 616–619 (2020). 33361792 10.1038/s41586-020-3030-1

[49] I. Amenabar, S. Poly, M. Goikoetxea, W. Nuansing, P. Lasch, R. Hillenbrand, Hyperspectral infrared nanoimaging of organic samples based on Fourier transform infrared nanospectroscopy. Nat. Commun. 8, 14402 (2017). 28198384 10.1038/ncomms14402 PMC5316859

[50] M. Schnell, M. Goikoetxea, I. Amenabar, P. S. Carney, R. Hillenbrand, Rapid infrared spectroscopic nanoimaging with nano-FTIR holography. ACS Photonics 7, 2878–2885 (2020).

