# Supplementary material for: Ultrafast anisotropic dynamics of hyperbolic nanolight pulse propagation
Source: Sci Adv. 2023 Aug 25;9(34):eadi4407. doi: 10.1126/sciadv.adi4407 (PMC10456838; doi:10.1126/sciadv.adi4407)
Supplement: Supplementary file 1 — Notes S1 to S5 Figs. S1 to S13 Legends for movies S1 to S4 References [file sciadv.adi4407_sm.pdf]

Science Advances
AAAS

Supplementary Materials for

Ultrafast anisotropic dynamics of hyperbolic nanolight pulse propagation

Xin Zhang *et al.*

Corresponding author: Xinliang Zhang, xlzhang@mail.hust.edu.cn, Peining Li, lipn@hust.edu.cn

Sci. Adv. **9**, eadi4407 (2023)
DOI: 10.1126/sciadv.adi4407

The PDF file includes:

Notes S1 to S5
Figs. S1 to S13
Legends for movies S1 to S4
References

Other Supplementary Material for this manuscript includes the following:

Movies S1 to S4

Note S1. Bandpass sampling and reconstruction

Unlike conventional nano-FTIR experiments recording an interferogram at a fixed location, our high-dimensional spacetime nanoimaging scans the two-dimensional (2D) spatial distributions at various time delays. However, due to the nature of scanning probe microscopy, it is a time-consuming task to acquire a high-quality high-dimensional data set. In our experiments, measuring a range of 20 μm × 15 μm with 100 nm spatial resolution requires approximately 20 minutes. To expedite data acquisition, we employed the bandpass sampling strategy to reduce the amount of data in the time domain.

Figure S3A shows the spectrum of the illuminating pulse on an Au sample as a reference. The spectral range of the pulse covers the Reststrahlen band (RB) of calcite and primarily responds from 1000 cm^{-1} to 2000 cm^{-1}. A typical interferogram obtained from a fixed location on calcite is presented in fig. S3B. Amenabar et al. (*49*) pointed out that only half of the interferogram contains vibration information about the sample due to the causality. The polariton wave only appears at a positive time delay (i.e., on the left of the white light position), corresponding to the reference pulse being later than the scattered signal pulse. Hence, we recorded these data with $\tau \geq 0$, and the reconstructed spectrum could reflect the properties of polaritons.

According to the Nyquist criterion, the sampling frequency should be greater than twice the maximum frequency to acquire the time signal validly. In this experiment, the sampling frequency ω_s is determined by the mirror moving interval, which can be calculated using the equation $\omega_s=1/(2\Delta d)$, where Δd is the interval, and the factor of 2 is attributed to the round trip of the reference pulse. Satisfying Nyquist sampling frequency ($\omega_s \geq 4000$ cm^{-1}) requires $\Delta d \leq 1.25$ μm. Nevertheless, subsampling methods can be applied to sample time signals at a rate below the Nyquist criterion, such as bandpass sampling (*50*). The bandpass sampling operates only in particular frequency ranges and must satisfy the following conditions:

$$\frac{2\omega_H}{m+1} \leq \omega_s \leq \frac{2\omega_L}{m} \qquad \text{(S1)}$$
$$2BW \leq \omega_s$$

where ω_H is the highest frequency of the spectra (ω_H = 2000 cm^{-1} in our case), ω_L is the lowest frequency (ω_L = 1000 cm^{-1} in our case), BW is the bandwidth, and m is the spectral replication number. We found that ω_s = 2000 cm^{-1} with m = 1 was appropriate for our experiment. It corresponds to an interval of Δd = 2.5 μm for the reference mirror movement, producing a time delay interval of $\Delta\tau$ = 16.7 fs. The comparison of an over-sampled spectrum (green line) and the bandpass sampled case (orange circles) is shown in fig. S3C. The resulting bandpass sampled spectral points are consistent with the over-sampled spectrum, indicating that our bandpass sampling strategy is capable of efficiently reconstructing the spectrum of calcite.

We interpolated the bandpass sampled data (fig. S1) to obtain a complete data set shown in movie S1 which shows the formation and propagation of HP pulses in space and time. To achieve this, we performed the Fourier transform (FT) processing to convert the high-dimensional spatiotemporal raw data into the frequency domain and retained the effective spectral range (1000 cm^{-1}–2000 cm^{-1}) with the conjugate spectra (0–1000cm^{-1}) removed. More details about the data preprocessing before FT are discussed in note S2. The data in the space-frequency domain could be transformed back to a spatiotemporal data set by the inverse FT. We applied a Tukey window with cosine fraction 1 for apodization and implemented the zero-padding by a factor of four in the frequency dimension to rectify the inadequate sampling rate. The zero-padded data reserves the unbroken spectral information and only interpolates due to the expanded sampling frequency.

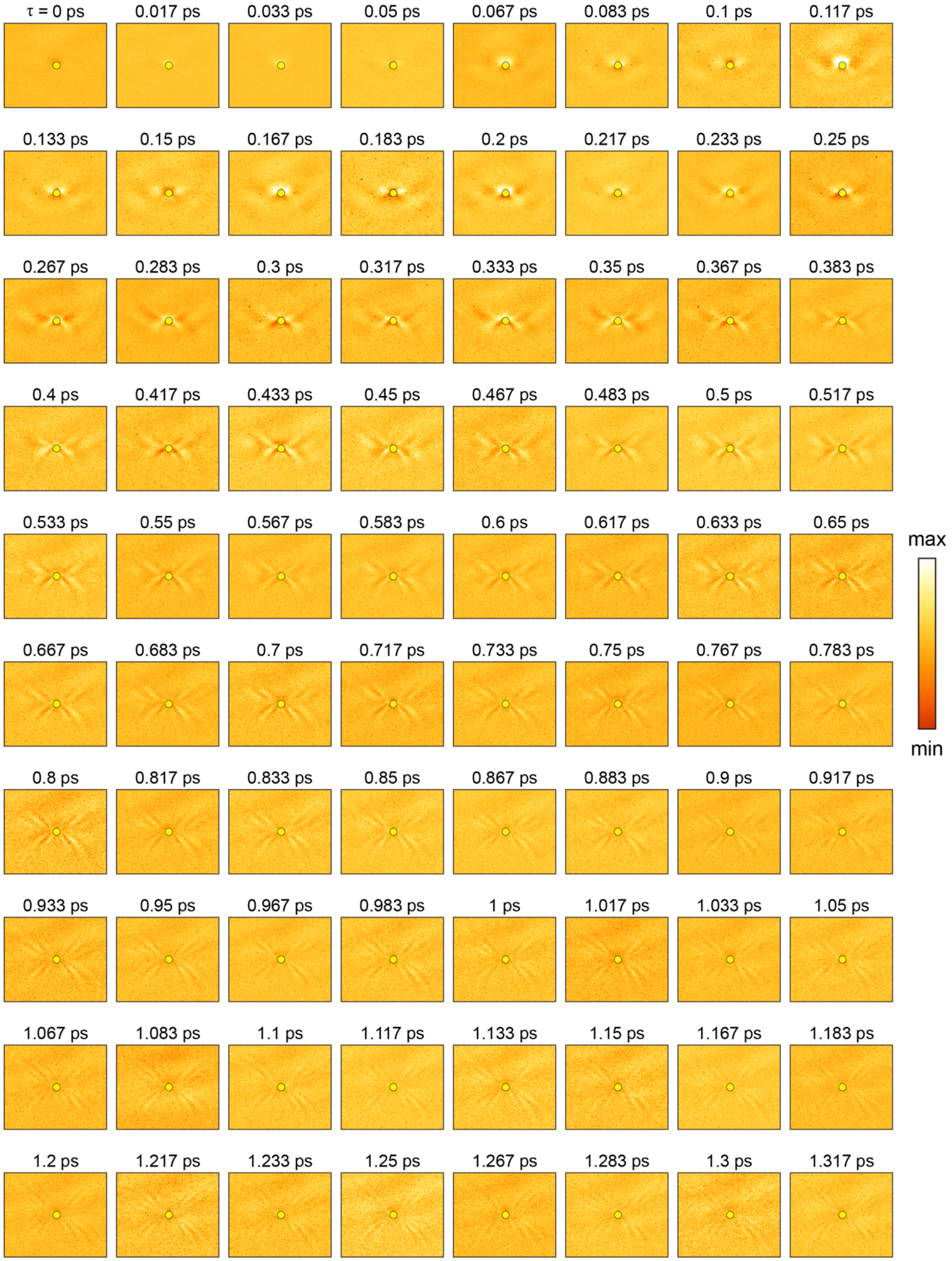

Fig. S1. Raw data of the time-domain interferometry nanoimaging. These images display experimental near-field amplitude snapshots (s_2), while the data processing was implemented with the complex-valued data.

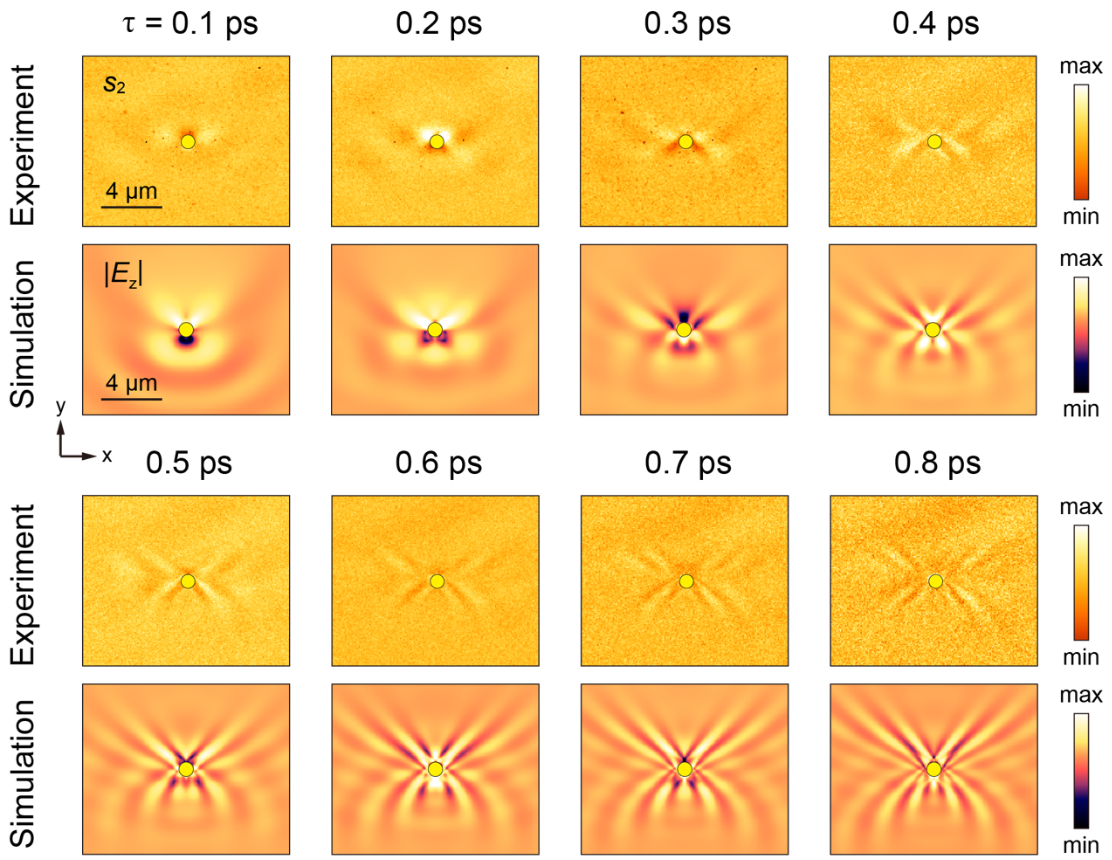

Fig. S2. Comparison of experimental spatiotemporal data with simulation results. Top panels, experimental near-field amplitude snapshots. Bottom panels, simulated near-field amplitude snapshots.

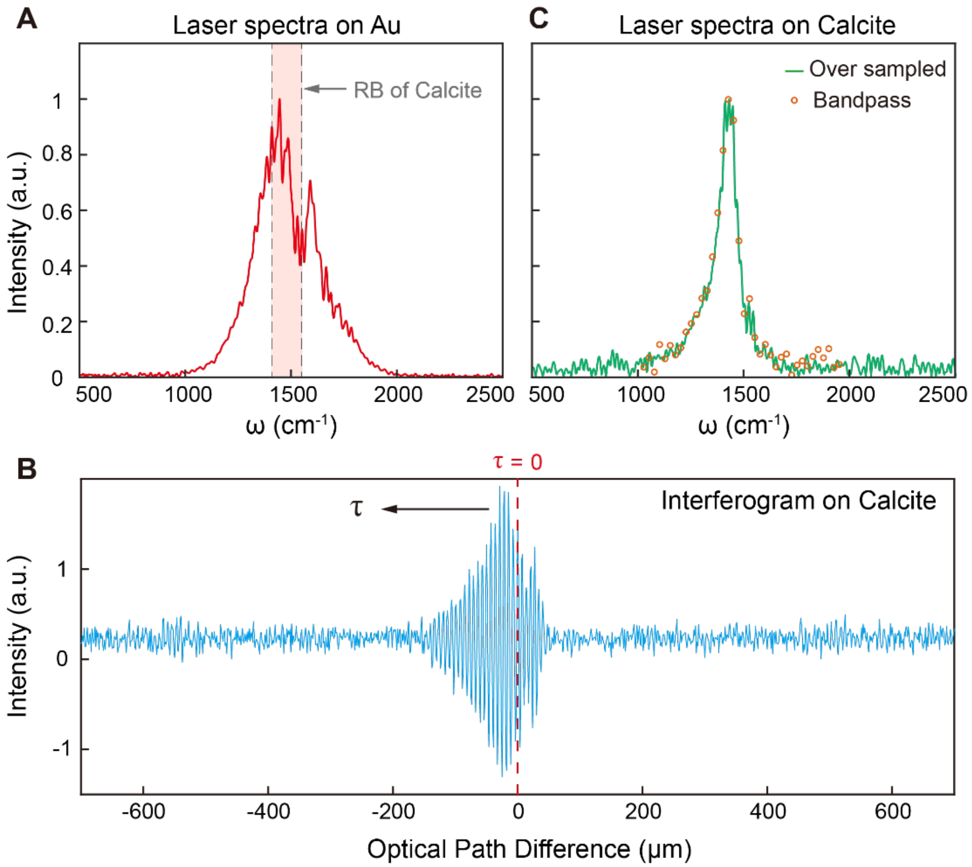

Fig. S3. Bandpass sampling reduces the number of time-domain interferogram points. (**A**) Amplitude spectrum on a gold reference sample (red line) shows that the broad range of the mid-infrared pulse used in the experiment covers the Reststrahlen band of calcite (shaded red area). The pulse mainly responses starting from 1000 cm^{-1} to 2000 cm^{-1}, which could be reconstructed by a bandpass sampling method. RB, Reststrahlen band. (**B**) A typical interferogram for calcite. The arrow shows the positive time variation direction, which means the reference pulse is later than the scattered signal pulse. (**C**) Amplitude spectra on the calcite sample. The green line shows the spectrum transformed from an interferogram with a total length of 350 μm (i.e., 700 μm optical path difference) and intervals of 0.625 μm (corresponding to an oversampling rate). Orange circles present spectral points transformed from an interferogram with a total length of 200 μm and intervals of 2.5 μm (corresponding to the bandpass sampling rate). These interferograms come from the left of the white light position (where the optical path difference is zero). These orange circles are consistent with the green line, confirming that our bandpass sampling strategy could efficiently reconstruct the spectrum of calcite based on reduced interferogram points. It also proves that our total sampling length of 200 μm in the high-dimensional spacetime nanoimaging experiment is adequately convergent for calcite.

Note S2. Extracting the background-free HP pulse

Discussion of interferometric signals

The time-domain interferometry records the interference between tip-scattered signals and reference pulse signals, which is illustrated in Fig. 1A of the main text. The detector's signal could be described by

$$E(x,y,\tau) \propto \int | E_{\rm sca}(x,y,t) + E_{\rm ref}(x,y,t-\tau) |^2 \, {\rm d}t \qquad \text{(S2)}$$

where x, y are the position coordinates, t is time, τ is the time delay, $E_{\rm ref}$ represents the reference signal, and $E_{\rm sca}$ is the tip-scattered signal that could be divided into two parts, i.e., $E_{\rm sca} = E_{\rm dir} + E_{\rm nf}$, where $E_{\rm dir}$ represents the illuminating field scattered directly by the tip and $E_{\rm nf}$ represents the polariton near-field distribution.

After demodulating the detector's signal at a higher harmonic $n\Omega$ ($n \geq 2$), according to Yoxall et al. (*6*), the detector's signal could be rewritten to

$$E_n(x,y,\tau) \propto \int (E_{n,\rm NI} + E_{n,\rm dir}E^*_{\rm ref} + E_{n,\rm nf}E^*_{\rm ref} + c.c.){\rm d}t \qquad \text{(S3)}$$

where $E_{n,\rm NI}$ is the self-homodyne term, and *c.c.* represents the complex conjugate of cross-terms. In this paper, we used the signals demodulated at two harmonics and left out the index n in the following for simplicity.

Inspired by the references (*6, 29*), we developed a data processing method to extract the intrinsic near-field distributions of the hyperbolic polariton pulse from interferometric signals. The non-interferometric component $E_{\rm NI}$ is a zero-frequency component that could be effectively removed in the frequency domain. The cross-correlation term $E_{\rm dir}E^*_{\rm ref}$ contributes to the zero-momentum component, which could be eliminated partly by the baseline subtraction processing performed in the references (*6, 29*). We proposed a further refinement utilizing Fourier space filtering to eliminate the noise in the momentum domain.

Preprocessing before Fourier space filtering

In the experiment, we measured a 20 μm × 15 μm area at each time delay with a 100 nm spatial resolution. The optical signal and the topography height were collected in the scanning procedure simultaneously, and the topography image was more stable for the identification of the disk's location. We centered the disk in each topography map and tailored the research area to a smaller one of 17 μm × 14 μm expanding from the reference origin (fig. S4A). Subsequently, we cropped near-field images according to adjusted regions (fig. S4B), producing an image of 170×140 pixels at each time delay. This processing could offer advantages for removing dirty regions and correcting the sample drift caused by repeated 2D scans.

To remove the undesired background, we performed the baseline subtraction processing. Specifically, we calculated the average value in a region far from the disk (green square in fig. S4B) as the background value for each near-field image. This area covers 170×10 pixels, and polariton waves are absent. Then the background value was subtracted for each image, which could efficiently reduce the zero-momentum component and facilitate the subsequent 2D FT processing. We also excluded the data where the disk was located by setting them to zero because they would contribute additional background in the 2D momentum domain.

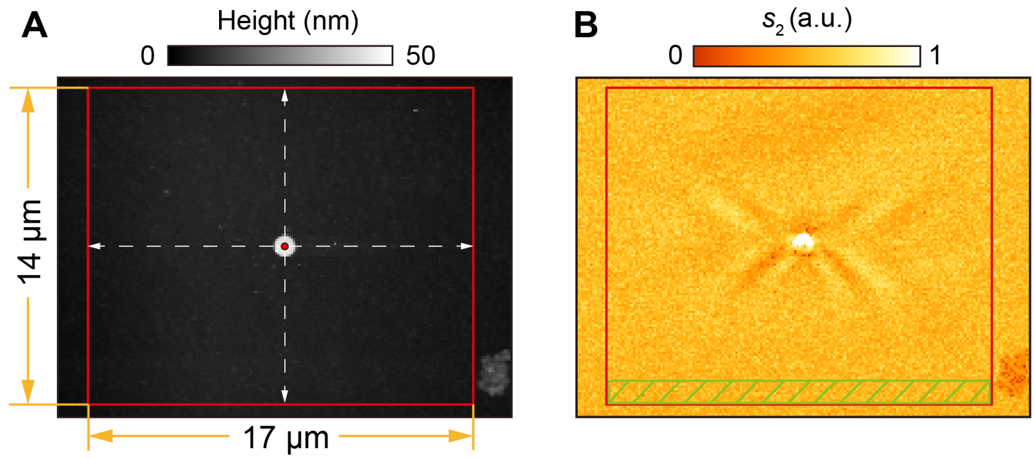

Fig. S4. Sample drift correction and tailoring. (**A**) Topography image of the scanning region. The whole region is up to 20 µm × 15 µm. We tailored an area of interest (red square, 17 µm × 14 µm) with the help of setting the disk center (red dot) as a reference origin. (**B**) Near-field image of this region at τ = 0.6 ps. The red square shows the research area corresponding to (A). The green square region with oblique lines presents the data range where we extracted the value of baseline subtraction.

High-dimensional Fourier transform filtering

We illustrated the flow of our high-dimensional Fourier transform filtering methods in fig. S5. While only the absolute values or real part of these data cubes were displayed, the data processing was implemented with the complex-valued data.

Firstly, we performed the FT along the time axis to convert the preprocessed spatiotemporal data into the space-frequency domain (fig. S5B). Prior to the FT procedure, the space-time domain data was overlaid by a window function (one half of a Tukey window with cosine fraction 0.3) for apodization and zero-padded by a factor of eight for interpolation in the time axis. The processing was also applied in the bandpass reconstruction program mentioned in note S1. There are atypical patterns in the frequency range out of the Reststrahlen band (see mappings below $\omega = 1400\ cm^{-1}$), which differ from hyperbolic polaritons. In this section, we focus on these hyperbolic polariton modes, consequently removing these modes existing out of the RB. Additionally, we note that there are few polariton fringes in frequencies higher than $1500\ cm^{-1}$ despite the end of RB extending to $1550\ cm^{-1}$ (see the mapping in $\omega = 1525\ cm^{-1}$) due to HPs cut off at frequencies below the longitudinal optical (LO) phonon frequency. As a result, for the sake of simplicity, we only retained distributions in the frequency range from $1410\ cm^{-1}$ to $1500\ cm^{-1}$ (the green shaded region in fig. S5B) to reconstruct the HP pulse and set others to zero.

Subsequently, we employed a further filtering step to cancel the noise from near-field distributions in the momentum domain for each frequency. We applied window functions (Tukey windows with cosine fraction 0.5) simultaneously along the x-axis and y-axis and padded zeros by a factor of four for each image before 2D FT processing (fig. S6). Next, we transformed each complex-valued hyperspectral mapping into respective momentum-domain via 2D FT (fig. S5C). We could observe high-momentum noises in mappings where the peaks of amplitudes present hyperbolic dispersion. We filtered out these noises via the 2D filtering processing shown in fig. S7 (taking the mapping in $\omega = 1450\ cm^{-1}$ as an example). Details of the procedure are described below:

1. Remove the parts whose amplitudes are lower than a threshold (1/e of the maximum could be an appropriate value) to highlight the hyperbolic dispersion.
2. Find peak points of the amplitude mapping in each row for four quadrants, respectively. Red circles in fig. S7A show these peak points that are five rows apart for better visualization.
3. Fit a hyperbola based on these peak points for each quadrant (see white dashed lines in fig. S7A). For frequencies lower than $1450\ cm^{-1}$, we fitted the hyperbola using points with $|k_y| > 0.2\pi\ \mu m^{-1}$ due to other low-k modes with elliptical dispersions existing in low frequencies, and we only wanted to fit the hyperbolic dispersion contour.
4. Take points in the fitted hyperbola as centers and generate a 2D filter function based on a Gaussian function of the full-width $\Delta k_x = 2\ \mu m^{-1}$ (fig. S7B). For the unfitted low-k ranges in Step 3, we used peak points as centers to preserve the low-k modes in low frequencies. We made the function width decrease gradually within $1.2\pi\ \mu m^{-1} < |k_y| < 1.6\pi\ \mu m^{-1}$ and set the width to zero when $|k_y| > 1.6\pi\ \mu m^{-1}$ to let the contour be smooth in the corner.
5. Apply filter functions to the momentum-domain mapping and produce the filtered hyperbolic dispersion contour (fig. S7C).

After filtering the momentum-domain mapping for each frequency, the noise-cancelled data cube was obtained as shown in fig. S5D. Performing the 2D inverse Fourier transform (iFT), we registered the data into the space-frequency domain again (fig. S5E). We still applied Tukey window functions and quadruple zero-padding as the preprocessing before 2D iFT. Finally, we transformed filtered hyperspectral imaging results to the space-time domain via iFT with a

quadruple zero-padding. Figure S5F shows the noise-cancelled intrinsic HP pulse distributions without non-hyperbolic dispersion frequency components. We analyzed the anisotropic spatiotemporal dynamics of HP pulse propagation based on the filtered spatiotemporal data.

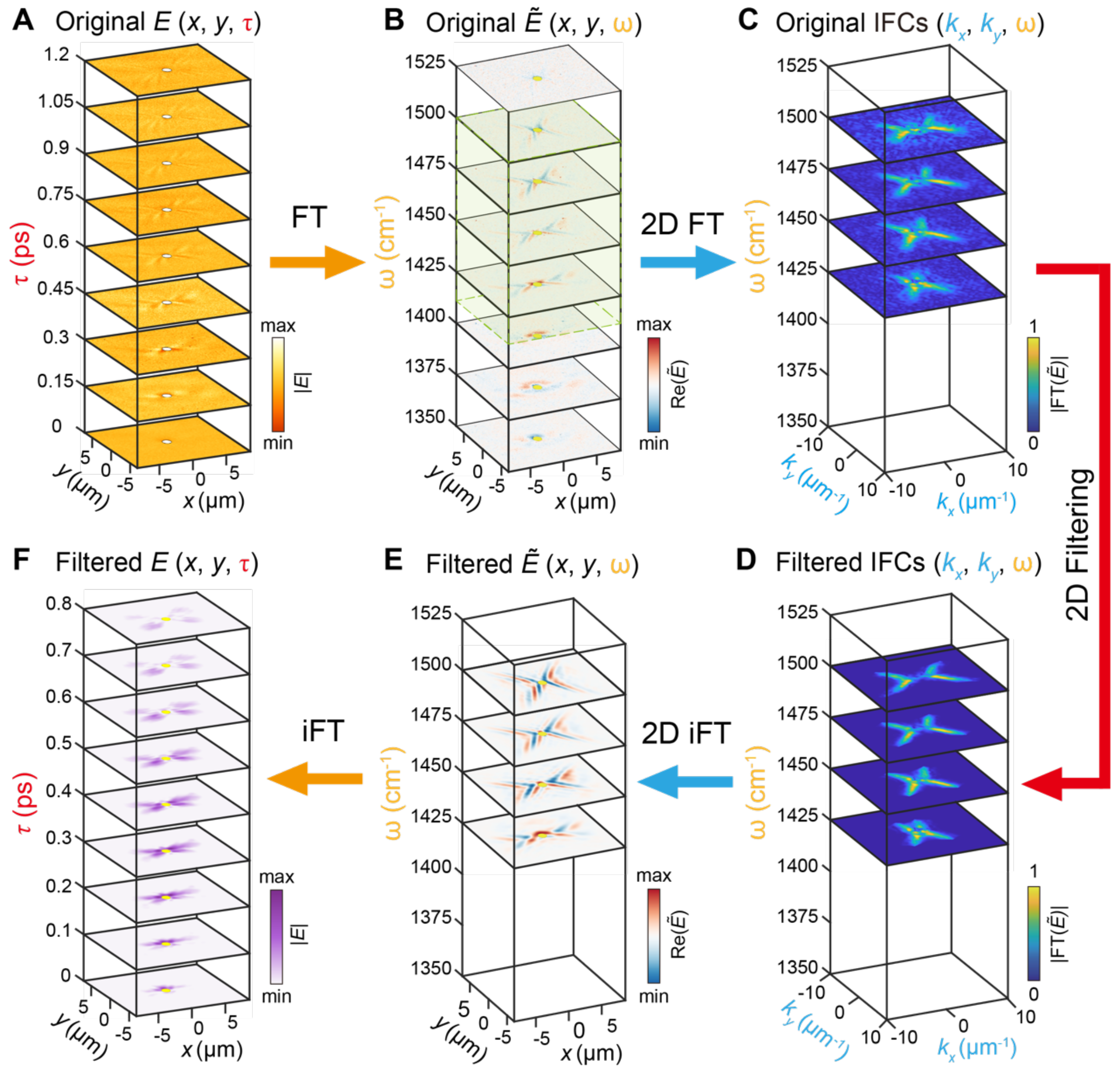

Fig. S5. Extracting background-free HP pulse fields. (**A**) Experimental raw data. (**B**) Space-frequency domain mappings (i.e., hyperspectral nanoimaging) are transformed from the data shown in (A) via FT. The green shaded region shows the frequency range where HPs exist. (**C**) Wavevector-frequency domain mappings (i.e., isofrequency contour) are transformed from the data shown in (B) via 2D FT. We only process these frequencies for HP pulse reconstruction. (**D**) IFCs are filtered from the data shown in (C) via the 2D filtering process. (**E**) Filtered space-frequency domain mappings are transformed from the data shown in (D) via 2D iFT. (**F**) Background-free space-time domain mappings are transformed from the data shown in (E) via iFT, which show the intrinsic HP pulse near-field distributions. Note that here we showed the absolute value or real part of the data cube, while the data processing was implemented with the complex-valued data. FT, Fourier transform; iFT, inverse Fourier transform.

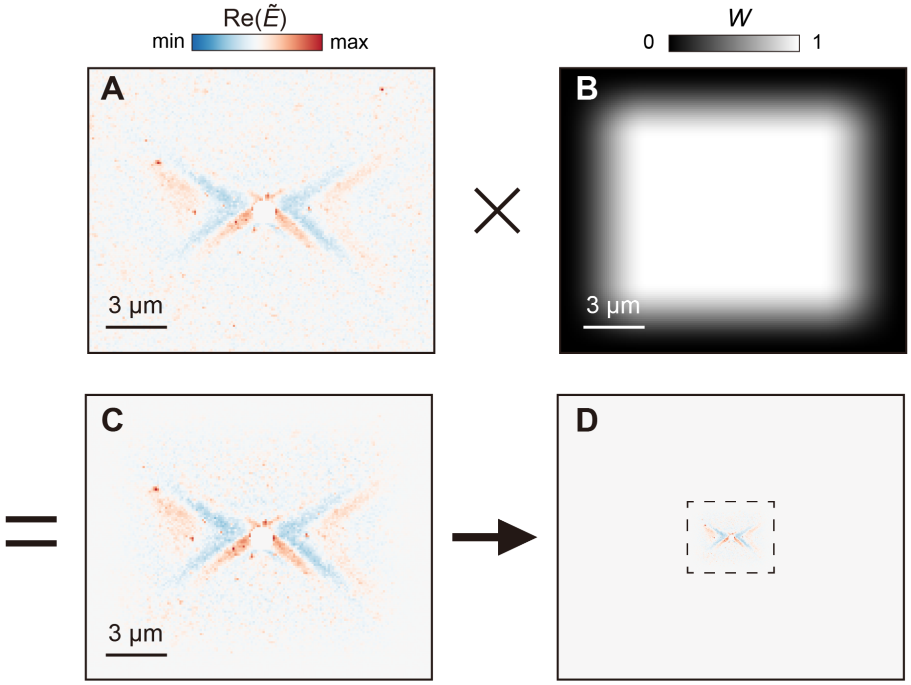

Fig. S6. Preprocessing before 2D FT. (**A**) Origin mapping (real part) in the space-frequency domain at 1450 cm^{-1}. (**B**) 2D window function. (**C**) Mapping after applying the window function. (**D**) Mapping after zero-padding. The dashed square shows the range in (C). We took the 2D transform in the space-frequency domain as an example, but the same preprocessing would be applied to other 2D FT processing.

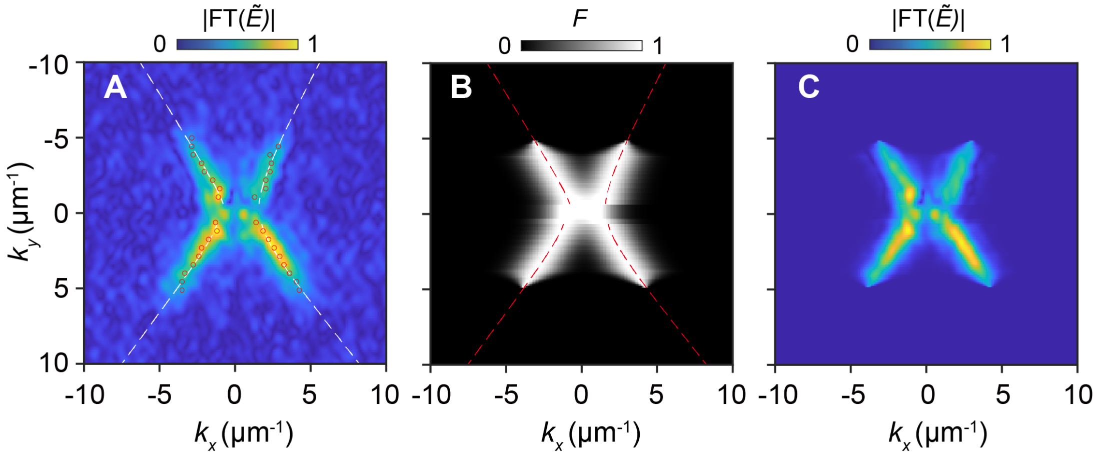

Fig. S7. Two-dimensional Fourier space filtering. (**A**) Absolute value of the *k*-space mapping at 1450 cm^{-1}. Red circles show the peak points of the mapping in each row, giving rising to the fitted hyperbola (white dashed lines). (**B**) 2D filter function. Red dashed lines represent the fitted hyperbola. (**C**) Absolute value of the filtered *k*-space mapping after applying the window function.

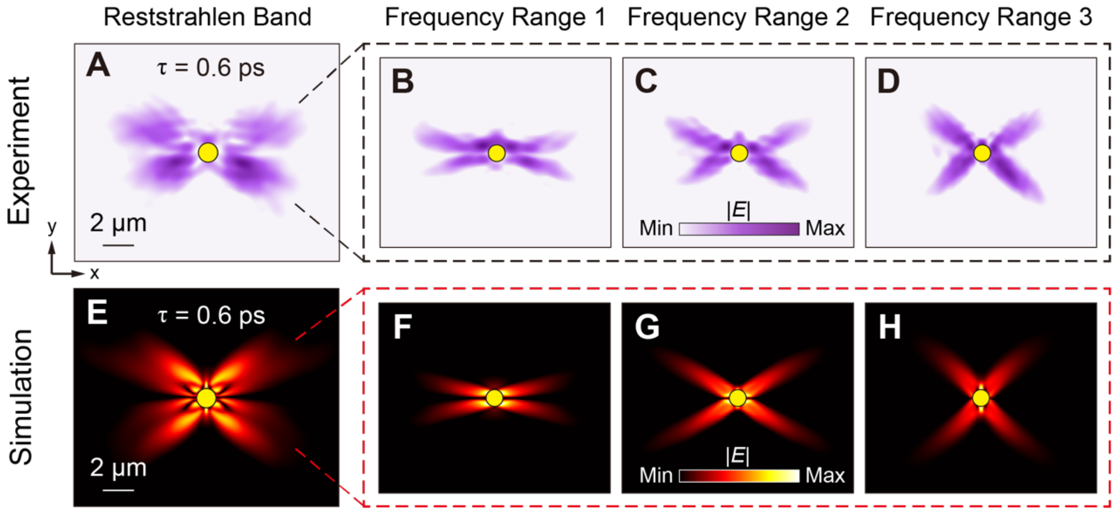

Fig. S8. Beam spreading of HP pulses. (**A**) Experimental near-field amplitude snapshot of HP pulses at $\tau = 0.6$ ps. It can be decomposed into different frequency components of HP pulse fields: (**B**) 1410–1440 cm^{-1}, (**C**) 1440–1470 cm^{-1}, and (**D**) 1470–1500 cm^{-1}. Note that the decomposed frequency ranges are arbitrarily chosen. The different spatiotemporal distributions are obtained by the same Fourier transform filtering processing discussed in note S2, but only changing the processed frequency range from the whole Reststrahlen band to three separate narrow frequency ranges. (**E** to **H**), Simulation results corresponding to A to D. When HP pulses are excited, the sequent emergence from the low-frequency to high-frequency component gives rise to the beam spreading and anomalous curved trajectories.

Note S3. Analysis of anisotropic energy dissipations

We performed linear interpolation for the absolute value of the filtered high-dimensional spacetime data set (shown in Fig. 2E) in a chosen angle α to generate a cross-section slice shown in Fig. 3C. The slice angle α is illustrated by the top-right inset in Fig. 3C (see also fig. S9), and the coordinate origin is relative to the disk center. For each amplitude cross-section slice, we extracted a contour line of the 1/e value of the maxima of the overall beam in the high-dimensional spacetime (see dashed lines in Fig. 3C and the purple surface in fig. S9). We derived the maximum time in the profile as the energy dissipation time τ_m. By analyzing the energy dissipation time in various directions, we could evaluate anisotropic energy dissipations of HPs.

Owing to the asymmetrical excitation produced by the oblique illumination, the forward disk extremity holds a brighter near-field hot spot (*12*), which brings the origin of the pulse fringes close to the disk edge than the center. For that reason, before extracting τ_m in different directions, we corrected the coordinate origin from the disk center to the forward disk edge in the direction of incidence. Subsequently, we extracted cross-section slices and obtain τ_m that are 5° apart within 0–180° to reveal anisotropic dissipations of HPs. It should be noted that there are no distinct pulse packet features in the cross-section slice with angles close to 90° due to limited hyperbolic isofrequency contours. As a result, we only showed these angles presenting explicit spatiotemporal packet features. We evaluated inherent anisotropic energy dissipations of HPs based on the dipole-simulated spatiotemporal data (the data in a circle area with a radius of 1 μm beneath the dipole is set to zero). The simulated dissipation time is similar to the corrected experimental one (Fig. 3D), and their maximum values all occur at about 45°.

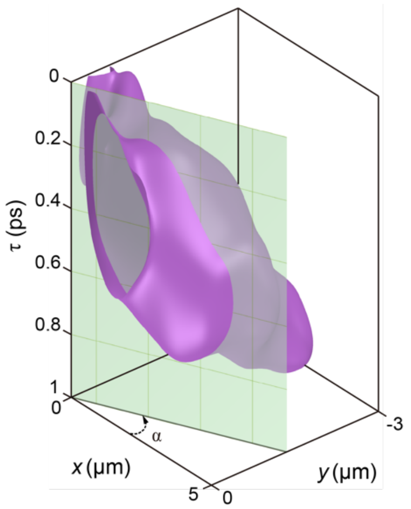

Fig. S9. Profile of one polariton beam in the high-dimensional spacetime. The purple surface indicates the profile where the pulse amplitude decay to 1/e of the maxima in this overall beam. The green plane illustrates the slice and α is the slice angle.

Note S4. Monitoring the time-dependent k-IFC

Notes for the *k-t* domain mapping results

We carried out 2D FT processing on the interpolated spatiotemporal data reconstructed in note S1 (movie S1) and acquired the *k-t* domain mapping shown in Fig. 4, A to H (see also movie S4). We applied Tukey window functions and eightfold zero-padding as the preprocessing before 2D FT. The data set comprises the entire spectra range of the incident pulse, in other words, including frequency components outside the RB of calcite. It is worth noting that the mapping in Fig. 4 (A to H) was transformed from the real part of the spatiotemporal data set (corresponding to the real signal). In the reconstruction procedure, we got complex-valued spatiotemporal data from the iFT processing.

Based on the disk-launched near-field distribution, which is shown in fig. S10 (A to C), we selected the disk as the coordinate origin to divide the 2D real space and executed the 2D FT processing for the complex-valued and real-valued 2D mapping, respectively. The resulting *k*-space mapping from 2D complex-valued data shows a package of *k*-components (fig. S10D), while the mapping from the real-valued data shows two branches in one quadrant (fig. S10G). The distinction is attributed to the nature of the fast Fourier transform algorithm, which generates a conjugated double-sided spectrum when applied FT on real-valued data, unlike a single-sided spectrum from complex-valued data. Consequently, one quadrant in the generated *k*-space mapping dominantly reflects the momentum component of one quadrant in real space when performing FT on complex-valued data. For instance, in fig. S10 (B, C, E and F), the first quadrant in *k*-space mainly reflects the momentum component in the fourth quadrant in real space, even including a diagonal distribution in the second real-space quadrant. However, this correspondence does not apply to the transform for real-valued 2D mapping. For this situation, the component of one quadrant in the *k*-space is affected by double-quadrant distributions in real space. In other words, polariton fields in diagonal real-space quadrants will contribute to the distribution in same *k*-space quadrants, yielding the interference phenomenon in *k*-space when analogous *k*-components overlay (see fig. S10, H and I). As a result, we ascribed the appearance of two branches in fig. S10G (see also Fig. 4H) to the data processing.

Figure S11 displays the *k-t* domain mapping transformed from complex-valued spatiotemporal data, in which we still observed the time-dependent topological transition for *k*-space IFCs. After this transition ($\tau \sim 0.25$ ps), the *k*-space mapping shows a packet of *k*-components meaning the presence of hyperbolic distributions with multiple frequencies at that time.

Extracting the largest wavevector of the IFC

To reveal the time dependence of the polariton field confinement, we selected one quadrant in *k*-space (the upper-right quadrant of mappings in Fig. 4, A to H) to extract the maximum wavevector values of the IFC. We took the 1/e value of the maximum amplitude as the threshold and plotted the contour line of this threshold (see black lines in fig. S12, B and D). We categorized the distribution out of the contour line to noises and calculated the maximum wavevector values inside the contour. Figure S12A shows the time-variation of the maximum polariton wavevector values from the data shown in Fig. 4, where a steeper slope can be observed around 0.25 ps corresponding to the transition from lenticular-shaped IFCs to hyperbolic ones. We also displayed the result obtained from the complex-valued spatiotemporal data in fig. S12C, which is in good agreement with the time-variation in fig. S12A, where the increased slope and approximate values are still present.

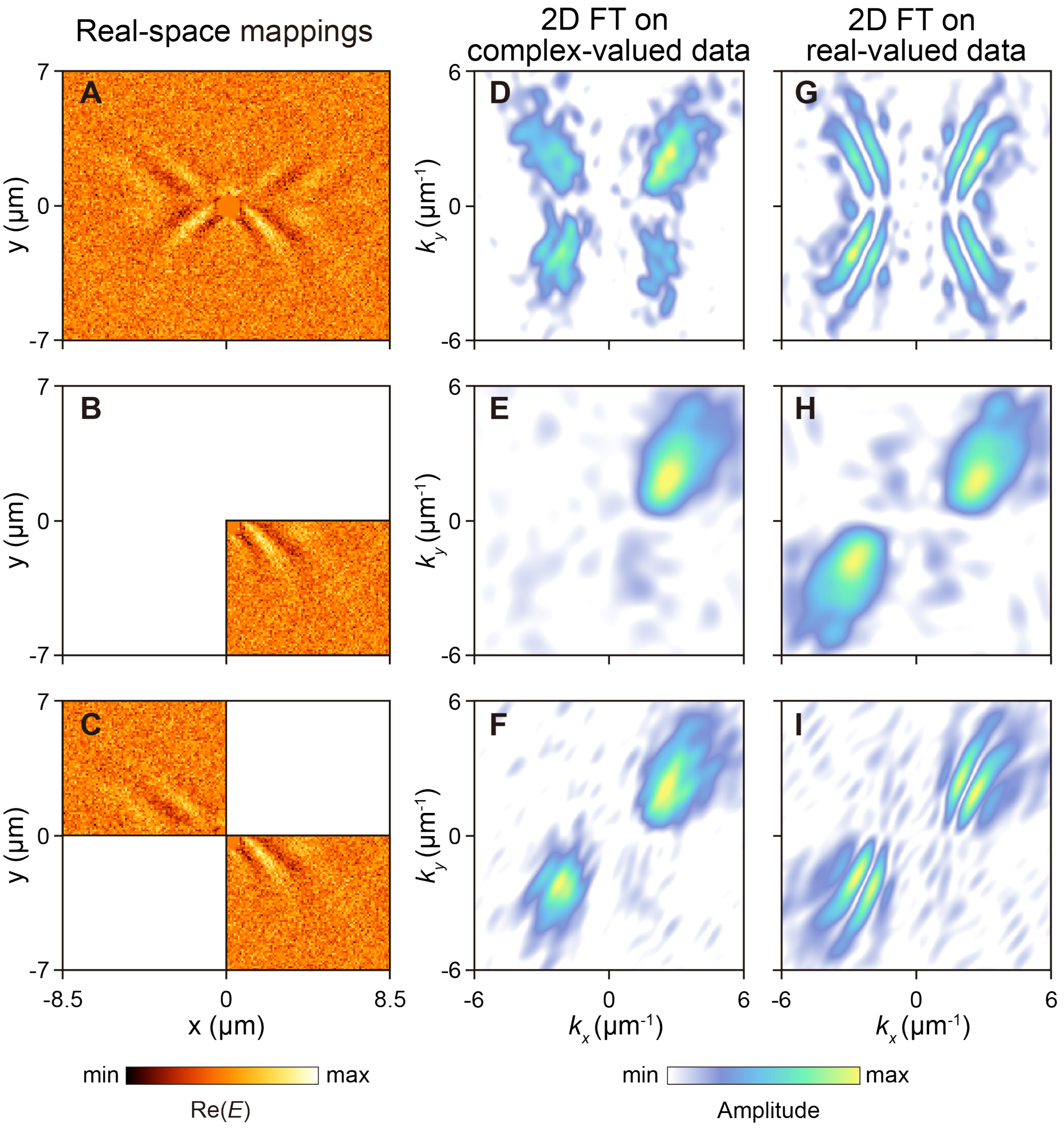

Fig. S10. Comparison of 2D FT results. (**A** to **C**) Real-space mappings at $\tau = 0.6$ ps with different clipping regions, where the data in white areas is removed. (**D** to **F**) Corresponding *k*-space amplitude mappings are obtained via 2D Fourier transform on complex-valued data in A (D), B (E) and C (F). (**G** to **I**) Corresponding *k*-space amplitude mappings are obtained via 2D Fourier transform on the real part of data in A (G), B (H) and C (I).

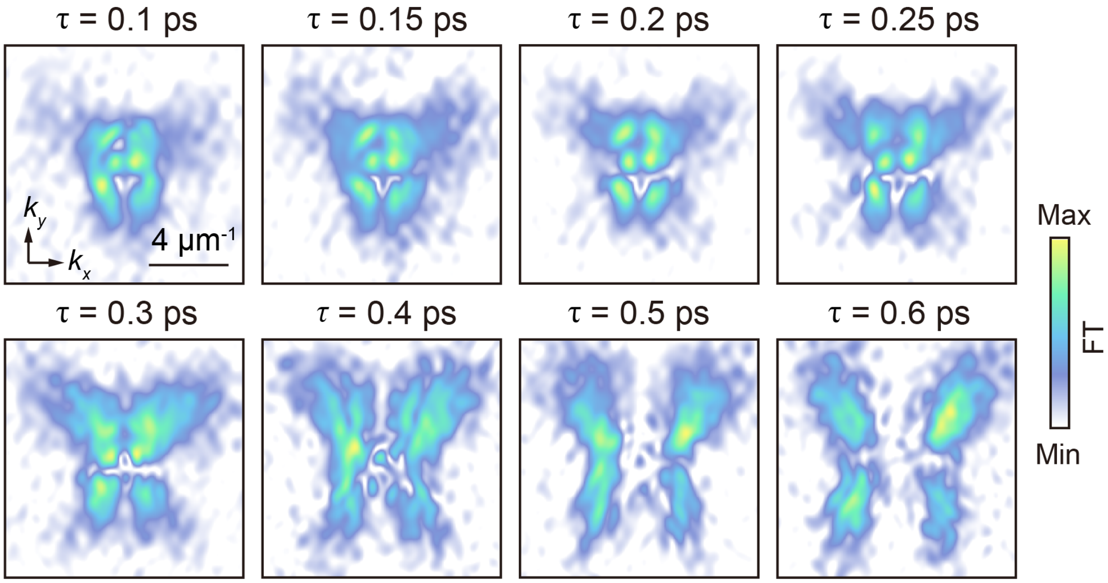

Fig. S11. Time-dependent *k*-space IFCs. Time-dependent *k*-space IFCs obtained by performing 2D FT on the complex-valued spacetime data. The topological transition occurs at around τ = 0.25 ps.

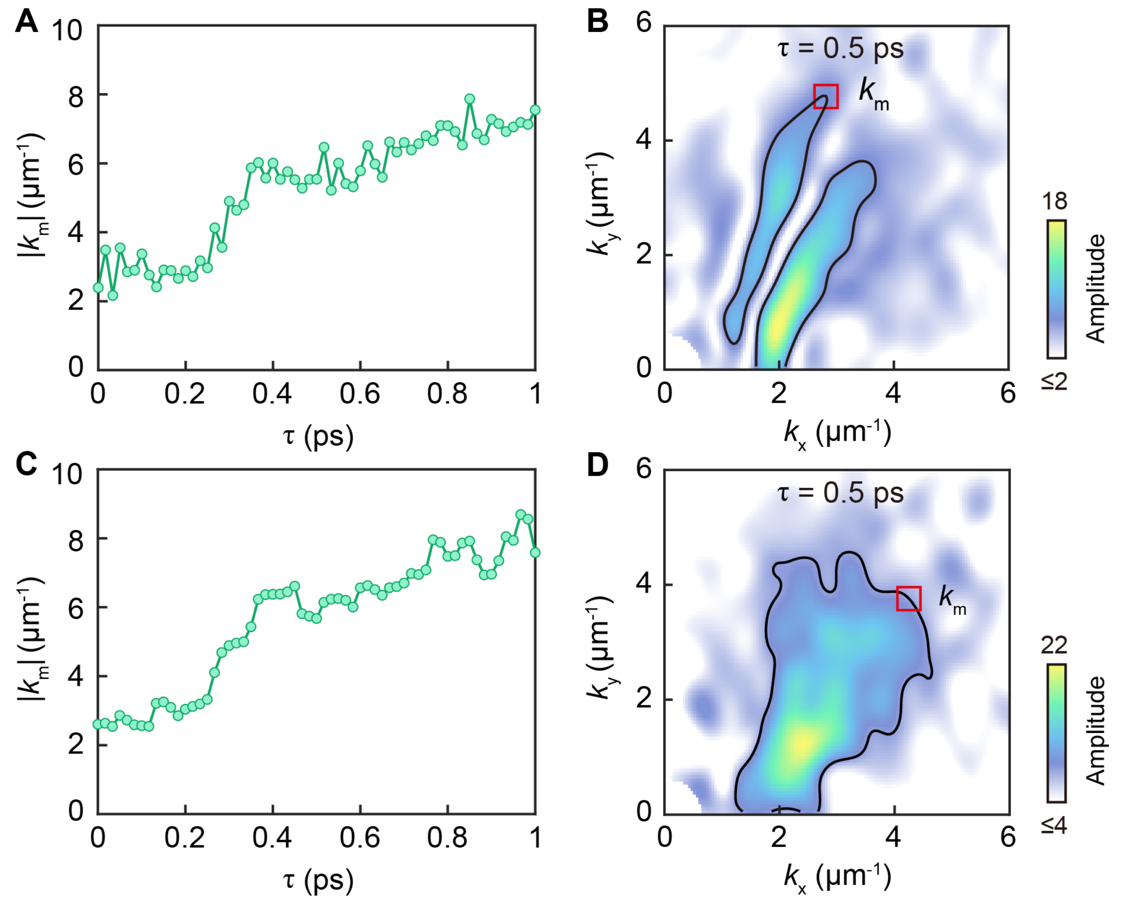

Fig. S12. Time-dependent *k*-space largest wavevectors. (**A**) Time-variation of the maximum wavevector k_m values derived from the time-dependent *k*-space IFCs (shown in Fig. 4, A to H) transformed from the real part of spacetime data. (**B**) Amplitude of IFCs in one quadrant at τ = 0.5 ps. The black line shows the contour line of 1/e of the maximum amplitude in this quadrant, which indicates the range of valid wavevector distributions. The red square marks the largest wavevector k_m. (**C**) and (**D**) Same as panels (A) and (B), respectively, but time-dependent *k*-space IFCs (shown in fig. S11) are derived from the complex-valued spacetime data.

Note S5. Analysis of the time-dependent momentum variation

In this section, we developed a theory to derive the momentum variation of the polariton pulse as a function of time using the high-dimensional time-momentum mappings obtained from our study. The momentum change of the polariton pulse could be attributed to two components, the generation of a growing momentum component and the propagation of pulses which brings about phase change in the momentum contribution. Hence, we first calculated the phase evolvement of polariton pulse propagation before deriving the momentum variation.

We considered a one-dimensional Gauss pulse propagating in a medium with group dispersion, which could be described by the equation

$$E(x,\tau) = \exp[-(\frac{x - v_g\tau}{\gamma})^2]\exp[i(k_0 x - \omega_0\tau)] \quad \text{(S4)}$$

where v_g is the group velocity, γ is the decay constant, ω_0 is the center frequency and k_0 is the wave vector relative to the center frequency. The distribution function in momentum space is

$$\begin{aligned} N(k,\tau) &= f(k)\exp[i\Phi(k,\tau)] \\ &= A_0 \exp[-\frac{1}{4}\gamma^2(k-k_0)^2]\exp\{i[v_g(k_0-k)-\omega_0]\tau\} \end{aligned} \quad \text{(S5)}$$

where A_0 is a scaling coefficient, $f(k)$ is the amplitude of momentum distribution (which is constant when excluding the generation of momenta), and $\Phi(k,\tau)$ is the phase distribution.

We could derive the phase increment at a time delay $\Delta\tau$ by

$$\Delta\Phi = [v_g(k_0 - k) - \omega_0]\Delta\tau \quad \text{(S6)}$$

For the two-dimensional pulse, it could be written as

$$\Delta\Phi = [\mathbf{v}_g(\mathbf{k}_0 - \mathbf{k}) - \omega_0]\Delta\tau \quad \text{(S7)}$$

For the ultraslow polariton pulse, the group velocity is much smaller that the light velocity, thus the first term in equation could be neglected. Therefore, we obtained the increment for each **k**-component by

$$\begin{aligned} \Delta N(\mathbf{k},\tau) &= N(\mathbf{k},\tau+\Delta\tau) - \alpha(\mathbf{k},\tau)N(\mathbf{k},\tau)\exp(i\Delta\Phi) \\ &\approx N(\mathbf{k},\tau+\Delta\tau) - \alpha N(\mathbf{k},\tau)\exp(-i\omega_0\Delta\tau) \end{aligned} \quad \text{(S8)}$$

where $N(\mathbf{k},\tau)$ is the wavevector distribution in our experimental time-momentum mappings, and α is the decay factor assessed by the whole momentum change. We obtained $\alpha \sim 0.99$ and ω_0 = 1445 cm^{-1} from our experiment results.

For each time delay, we could calculate the wavevector increment by

$$\Delta\mathbf{k} = \iint |\Delta N| \mathbf{k} \mathrm{d}k_x \mathrm{d}k_y \quad \text{(S9)}$$

An appropriate filter processing was required to extract the polariton momentum distribution from the background. We found the near-field distribution of pulses was present in the range of 1300 cm^{-1}–1500 cm^{-1}. As a result, we retained the distributions within this frequency range while filtering out all others. Within the frequency range of 1400 cm^{-1}–1500 cm^{-1}, a two-dimensional Fourier space filtering was applied (consistent with the process in note S2). Within the frequency range of 1300 cm^{-1}–1400 cm^{-1}, we filtered the data set in the frequency-momentum domain by eliminating the components whose amplitudes were lower than 1/e of the maximum for each frequency. The filtered data was transformed into the space-time domain and then analyzed to determine the momentum increment $\Delta\mathbf{P} = \hbar\Delta\mathbf{k}$. The momentum increment at τ = 0.2 ps is illustrated in Fig. 4I in the main text as an example.

According to the linear momentum conservation law, the optical force exerted on the disk could be relative to the momentum change of polariton pulses and calculated by summing the changes in momentum for all wavevector components using the equation of

$$\mathbf{F}(\tau) = -\frac{\sum \Delta \mathbf{P}}{\Delta \tau} = -\frac{\hbar \sum \Delta \mathbf{k}}{\Delta \tau} = -\frac{\iint |\Delta N| \hbar \mathbf{k} \mathrm{d}k_{\mathrm{x}} \mathrm{d}k_{\mathrm{y}}}{\Delta \tau} \tag{S10}$$

where $\hbar$ is the reduced Plank constant.

To obtain an estimated value of the optical force, we rescaled the momentum distribution by taking into account the coefficient induced by the measurement and data processing. In the experiment, we used the pulse laser with a power of P_0 = 0.5mW and a repetition frequency of M = 80MHz. Pons-Valencia et al. (*40*) reported the max launching efficiency of hBN polaritons by the gold resonant antenna is up to 0.1 but is smaller for non-resonant frequencies, so we assumed the transfer efficiency from the laser pulse to the polariton pulse is η = 1% for our experiments. Then the polariton pulse energy in one laser pulse cycle could be calculated by $E_0 = \eta P_0/M$. Besides, we roughly calculated the pulse energy from our time-momentum data by

$$E_{\mathrm{p}} = c \iint |N(\mathbf{k}, \tau_0)| \hbar \mathbf{k} \mathrm{d}k_{\mathrm{x}} \mathrm{d}k_{\mathrm{y}} \tag{S11}$$

where c is the light velocity, and τ_0 is the time corresponding to the appearance of the max total momentum ($\tau_0 \sim$ 0.2ps). By comparing E_{p} and E_0, we rescaled the value of $|N(\mathbf{k},\tau_0)|$, thus we estimated the maximum transient time-dependent optical force **F** to be on the order of about 100 pN using the equation S10.

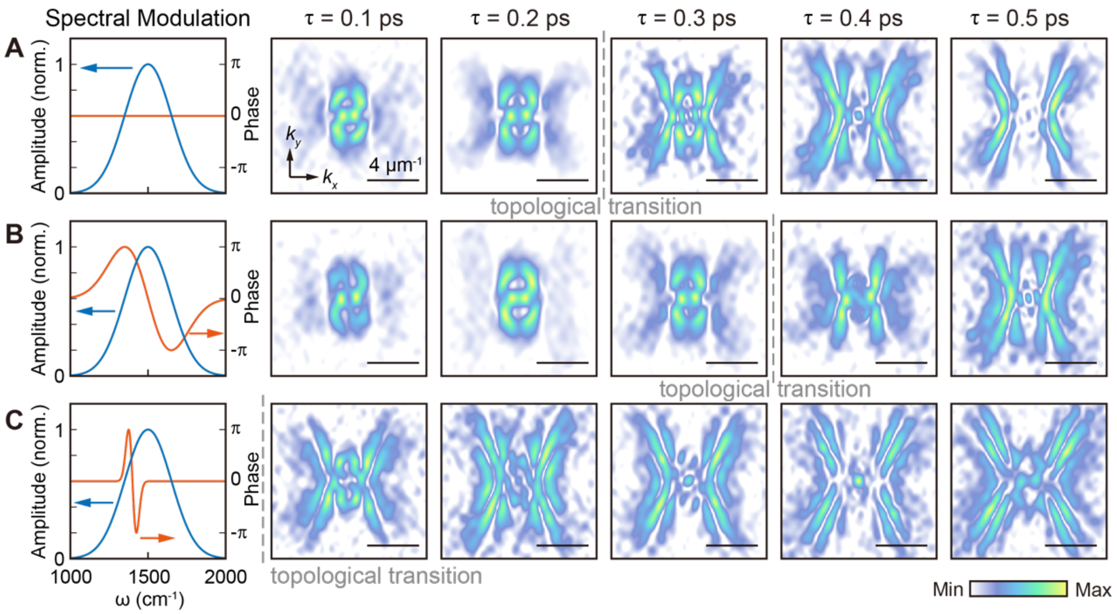

Fig. S13. Modulation of the time-domain topological transition. The polariton pulse could be modulated through the initial spectrum, resulting in distinctive topological transition characteristics in the time-momentum domain. To model the initial-spectral-phase-manipulated time-domain topological transition, we reconstructed different spatiotemporal data by adding various spectral phase modulations to the experimental space-frequency data, resulting in the transformed spatiotemporal data presenting different topological transition time and momentum distributions in the time-momentum domain. Specifically, we considered three cases: (**A**) Absence of additional phase modulation corresponding to actual experimental results. (**B**) A mild phase modulation in the overall laser spectrum but roughly linear in the range of 1300 cm^{-1}–1500 cm^{-1}, bringing about a shift in the topological transition time. (**C**) A pronounced phase modulation within 1300 cm^{-1}–1500 cm^{-1} leads to the alteration of the topological transition time and modification to momentum distributions.

Legends for movies S1 to S4

Movie S1. Ultrafast nano-optical movies of HP pulse propagation. It shows the complete evolution of HP pulses reconstructed via data interpolation (see note S1).

Movie S2. Spacetime evolutions of HP pulse velocity vectors. Small colored arrows show local distributions of fringe velocity $\mathbf{v}_f$, with the colors showing the real part of the near fields of HPs. The large pale gold arrows indicate the centroids of four HP beams, respectively. Their directions are aligned with the corresponding centroid velocity $\mathbf{v}_c$.

Movie S3. Spacetime variations of HP pulses energy. (**A**) time-dependent variations of background-subtracted HP pulse amplitudes. Black circles mark the centroid position for each HP beam, and the pale gold arrows indicate the centroid velocity direction. For comparison, panel (**B**) shows variations of the real part of HP pulse fields, which illustrate the phase variations of HP pulse fields. Blue arrows reveal the local distribution of the fringe velocities.

Movie S4. Spacetime movies of HP wavevectors. It reveals the time-dependent topological transition of HP wavevectors.

REFERENCES AND NOTES

1. R. W. Boyd, D. J. Gauthier, Controlling the velocity of light pulses. *Science* **326**, 1074–1077 (2009).

2. K. L. Tsakmakidis, O. Hess, R. W. Boyd, X. Zhang, Ultraslow waves on the nanoscale. *Science* **358**, eaan5196 (2017).

3. H. Gersen, T. J. Karle, R. J. Engelen, W. Bogaerts, J. P. Korterik, N. F. van Hulst, T. F. Krauss, L. Kuipers, Direct observation of Bloch harmonics and negative phase velocity in photonic crystal waveguides. *Phys. Rev. Lett.* **94**, 123901 (2005).

4. G. M. Gehring, A. Schweinsberg, C. Barsi, N. Kostinski, R. W. Boyd, Observation of backward pulse propagation through a medium with a negative group velocity. *Science* **312**, 895–897 (2006).

5. G. Dolling, C. Enkrich, M. Wegener, C. M. Soukoulis, S. Linden, Simultaneous negative phase and group velocity of light in a metamaterial. *Science* **312**, 892–894 (2006).

6. E. Yoxall, M. Schnell, A. Y. Nikitin, O. Txoperena, A. Woessner, M. B. Lundeberg, F. Casanova, L. E. Hueso, F. H. L. Koppens, R. Hillenbrand, Direct observation of ultraslow hyperbolic polariton propagation with negative phase velocity. *Nat. Photonics* **9**, 674–678 (2015).

7. R. W. Boyd, *Nonlinear Optics* (Academic Press, 2020).

8. S. Dai, Z. Fei, Q. Ma, A. S. Rodin, M. Wagner, A. S. McLeod, M. K. Liu, W. Gannett, W. Regan, K. Watanabe, T. Taniguchi, M. Thiemens, G. Dominguez, A. H. Castro Neto, A. Zettl, F. Keilmann, P. Jarillo-Herrero, M. M. Fogler, D. N. Basov, Tunable phonon polaritons in atomically thin van der Waals crystals of boron nitride. *Science* **343**, 1125–1129 (2014).

9. P. Li, I. Dolado, F. J. Alfaro-Mozaz, F. Casanova, L. E. Hueso, S. Liu, J. H. Edgar, A. Y. Nikitin, S. Velez, R. Hillenbrand, Infrared hyperbolic metasurface based on nanostructured van der Waals materials. *Science* **359**, 892–896 (2018).

10. W. Ma, P. Alonso-Gonzalez, S. Li, A. Y. Nikitin, J. Yuan, J. Martin-Sanchez, J. Taboada-Gutierrez, I. Amenabar, P. Li, S. Velez, C. Tollan, Z. Dai, Y. Zhang, S. Sriram, K. Kalantar-Zadeh, S. T. Lee, R.

Hillenbrand, Q. Bao, In-plane anisotropic and ultra-low-loss polaritons in a natural van der Waals crystal. *Nature* **562**, 557–562 (2018).

11. Z. Zheng, N. Xu, S. L. Oscurato, M. Tamagnone, F. Sun, Y. Jiang, Y. Ke, J. Chen, W. Huang, W. L. Wilson, A. Ambrosio, S. Deng, H. Chen, A mid-infrared biaxial hyperbolic van der Waals crystal. *Sci. Adv.* **5**, eaav8690 (2019).

12. W. Ma, G. Hu, D. Hu, R. Chen, T. Sun, X. Zhang, Q. Dai, Y. Zeng, A. Alù, C. W. Qiu, P. Li, Ghost hyperbolic surface polaritons in bulk anisotropic crystals. *Nature* **596**, 362–366 (2021).

13. N. C. Passler, X. Ni, G. Hu, J. R. Matson, G. Carini, M. Wolf, M. Schubert, A. Alù, J. D. Caldwell, T. G. Folland, A. Paarmann, Hyperbolic shear polaritons in low-symmetry crystals. *Nature* **602**, 595–600 (2022).

14. G. Hu, W. Ma, D. Hu, J. Wu, C. Zheng, K. Liu, X. Zhang, X. Ni, J. Chen, X. Zhang, Q. Dai, J. D. Caldwell, A. Paarmann, A. Alù, P. Li, C. W. Qiu, Real-space nanoimaging of hyperbolic shear polaritons in a monoclinic crystal. *Nat. Nanotechnol.* **18**, 64–70 (2023).

15. Y. Kurman, R. Dahan, H. H. Sheinfux, K. Wang, M. Yannai, Y. Adiv, O. Reinhardt, L. H. G. Tizei, S. Y. Woo, J. Li, J. H. Edgar, M. Kociak, F. H. L. Koppens, I. Kaminer, Spatiotemporal imaging of 2D polariton wave packet dynamics using free electrons. *Science* **372**, 1181–1186 (2021).

16. A. J. Sternbach, S. H. Chae, S. Latini, A. A. Rikhter, Y. Shao, B. Li, D. Rhodes, B. Kim, P. J. Schuck, X. Xu, X. Y. Zhu, R. D. Averitt, J. Hone, M. M. Fogler, A. Rubio, D. N. Basov, Programmable hyperbolic polaritons in van der Waals semiconductors. *Science* **371**, 617–620 (2021).

17. D. N. Basov, M. M. Fogler, F. J. Garcia de Abajo, Polaritons in van der Waals materials. *Science* **354**, aag1992 (2016).

18. T. Low, A. Chaves, J. D. Caldwell, A. Kumar, N. X. Fang, P. Avouris, T. F. Heinz, F. Guinea, L. Martin-Moreno, F. Koppens, Polaritons in layered two-dimensional materials. *Nat. Mater.* **16**, 182–194 (2017).

19. Q. Zhang, G. Hu, W. Ma, P. Li, A. Krasnok, R. Hillenbrand, A. Alù, C. W. Qiu, Interface nano-optics with van der Waals polaritons. *Nature* **597**, 187–195 (2021).

20. J. D. Caldwell, A. V. Kretinin, Y. Chen, V. Giannini, M. M. Fogler, Y. Francescato, C. T. Ellis, J. G. Tischler, C. R. Woods, A. J. Giles, M. Hong, K. Watanabe, T. Taniguchi, S. A. Maier, K. S. Novoselov, Sub-diffractional volume-confined polaritons in the natural hyperbolic material hexagonal boron nitride. *Nat. Commun.* **5**, 5221 (2014).

21. F. J. Rodriguez-Fortuno, G. Marino, P. Ginzburg, D. O'Connor, A. Martinez, G. A. Wurtz, A. V. Zayats, Near-field interference for the unidirectional excitation of electromagnetic guided modes. *Science* **340**, 328–330 (2013).

22. J. Lin, J. P. Mueller, Q. Wang, G. Yuan, N. Antoniou, X. C. Yuan, F. Capasso, Polarization-controlled tunable directional coupling of surface plasmon polaritons. *Science* **340**, 331–334 (2013).

23. A. J. Sternbach, S. L. Moore, A. Rikhter, S. Zhang, R. Jing, Y. Shao, B. S. Y. Kim, S. Xu, S. Liu, J. H. Edgar, A. Rubio, C. Dean, J. Hone, M. M. Fogler, D. N. Basov, Negative refraction in hyperbolic hetero-bicrystals. *Science* **379**, 555–557 (2023).

24. H. Hu, N. Chen, H. Teng, R. Yu, M. Xue, K. Chen, Y. Xiao, Y. Qu, D. Hu, J. Chen, Z. Sun, P. Li, F. J. G. de Abajo, Q. Dai, Gate-tunable negative refraction of mid-infrared polaritons. *Science* **379**, 558–561 (2023).

25. J. Duan, G. Alvarez-Perez, A. I. F. Tresguerres-Mata, J. Taboada-Gutierrez, K. V. Voronin, A. Bylinkin, B. Chang, S. Xiao, S. Liu, J. H. Edgar, J. I. Martin, V. S. Volkov, R. Hillenbrand, J. Martin-Sanchez, A. Y. Nikitin, P. Alonso-Gonzalez, Planar refraction and lensing of highly confined polaritons in anisotropic media. *Nat. Commun.* **12**, 4325 (2021).

26. J. Martin-Sanchez, J. Duan, J. Taboada-Gutierrez, G. Alvarez-Perez, K. V. Voronin, I. Prieto, W. Ma, Q. Bao, V. S. Volkov, R. Hillenbrand, A. Y. Nikitin, P. Alonso-Gonzalez, Focusing of in-plane hyperbolic polaritons in van der Waals crystals with tailored infrared nanoantennas. *Sci. Adv.* **7**, eabj0127 (2021).

27. H. Hu, N. Chen, H. Teng, R. Yu, Y. Qu, J. Sun, M. Xue, D. Hu, B. Wu, C. Li, J. Chen, M. Liu, Z. Sun, Y. Liu, P. Li, S. Fan, F. J. Garcia de Abajo, Q. Dai, Doping-driven topological polaritons in graphene/α-MoO_3 heterostructures. *Nat. Nanotechnol.* **17**, 940–946 (2022).

28. M. Autore, P. Li, I. Dolado, F. J. Alfaro-Mozaz, R. Esteban, A. Atxabal, F. Casanova, L. E. Hueso, P. Alonso-Gonzalez, J. Aizpurua, A. Y. Nikitin, S. Velez, R. Hillenbrand, Boron nitride nanoresonators for phonon-enhanced molecular vibrational spectroscopy at the strong coupling limit. *Light Sci. Appl.* **7**, 17172 (2018).

29. A. Bylinkin, M. Schnell, M. Autore, F. Calavalle, P. Li, J. Taboada-Gutierrez, S. Liu, J. H. Edgar, F. Casanova, L. E. Hueso, P. Alonso-Gonzalez, A. Y. Nikitin, R. Hillenbrand, Real-space observation of vibrational strong coupling between propagating phonon polaritons and organic molecules. *Nat. Photonics* **15**, 197–202 (2021).

30. S. Dai, Q. Ma, T. Andersen, A. S. McLeod, Z. Fei, M. K. Liu, M. Wagner, K. Watanabe, T. Taniguchi, M. Thiemens, F. Keilmann, P. Jarillo-Herrero, M. M. Fogler, D. N. Basov, Subdiffractional focusing and guiding of polaritonic rays in a natural hyperbolic material. *Nat. Commun.* **6**, 6963 (2015).

31. P. Li, M. Lewin, A. V. Kretinin, J. D. Caldwell, K. S. Novoselov, T. Taniguchi, K. Watanabe, F. Gaussmann, T. Taubner, Hyperbolic phonon-polaritons in boron nitride for near-field optical imaging and focusing. *Nat. Commun.* **6**, 7507 (2015).

32. F. Keilmann, R. Hillenbrand, Near-field microscopy by elastic light scattering from a tip. *Philos. Trans. A. Math. Phys. Eng. Sci.* **362**, 787–805 (2004).

33. T. L. Cocker, V. Jelic, R. Hillenbrand, F. A. Hegmann, Nanoscale terahertz scanning probe microscopy. *Nat. Photonics* **15**, 558–569 (2021).

34. X. Chen, D. Hu, R. Mescall, G. You, D. N. Basov, Q. Dai, M. Liu, Modern scattering-type scanning near-field optical microscopy for advanced material research. *Adv. Mater.* **31**, e1804774 (2019).

35. K. J. Tielrooij, N. C. H. Hesp, A. Principi, M. B. Lundeberg, E. A. A. Pogna, L. Banszerus, Z. Mics, M. Massicotte, P. Schmidt, D. Davydovskaya, D. G. Purdie, I. Goykhman, G. Soavi, A. Lombardo, K. Watanabe, T. Taniguchi, M. Bonn, D. Turchinovich, C. Stampfer, A. C. Ferrari, G. Cerullo, M. Polini,

F. H. L. Koppens, Out-of-plane heat transfer in van der Waals stacks through electron-hyperbolic phonon coupling. *Nat. Nanotechnol.* **13**, 41–46 (2018).

36. N. Rivera, I. Kaminer, Light-matter interactions with photonic quasiparticles. *Nat. Rev. Phys.* **2**, 538–561 (2020).

37. C. Hu, T. Sun, Y. Zeng, W. Ma, Z. Dai, X. Yang, X. Zhang, P. Li, Source-configured symmetry-broken hyperbolic polaritons. *eLight* **3**, 14 (2023).

38. T. Ozawa, H. M. Price, A. Amo, N. Goldman, M. Hafezi, L. Lu, M. C. Rechtsman, D. Schuster, J. Simon, O. Zilberberg, I. Carusotto, Topological photonics. *Rev. Mod. Phys.* **91**, 015006 (2019).

39. H. Li, Y. Cao, B. Shi, T. Zhu, Y. Geng, R. Feng, L. Wang, F. Sun, Y. Shi, M. A. Miri, M. Nieto-Vesperinas, C. W. Qiu, W. Ding, Momentum-topology-induced optical pulling force. *Phys. Rev. Lett.* **124**, 143901 (2020).

40. P. Pons-Valencia, F. J. Alfaro-Mozaz, M. M. Wiecha, V. Biolek, I. Dolado, S. Velez, P. Li, P. Alonso-Gonzalez, F. Casanova, L. E. Hueso, L. Martin-Moreno, R. Hillenbrand, A. Y. Nikitin, Launching of hyperbolic phonon-polaritons in h-BN slabs by resonant metal plasmonic antennas. *Nat. Commun.* **10**, 3242 (2019).

41. L. Xiong, Y. Li, D. Halbertal, M. Sammon, Z. Sun, S. Liu, J. H. Edgar, T. Low, M. M. Fogler, C. R. Dean, A. J. Millis, D. N. Basov, Polaritonic vortices with a half-integer charge. *Nano Lett.* **21**, 9256–9261 (2021).

42. M. Wang, G. Hu, S. Chand, M. Cotrufo, Y. Abate, K. Watanabe, T. Taniguchi, G. Grosso, C. W. Qiu, A. Alù, Spin-orbit-locked hyperbolic polariton vortices carrying reconfigurable topological charges. *eLight* **2**, 12 (2022).

43. N. Engheta, Four-dimensional optics using time-varying metamaterials. *Science* **379**, 1190–1191 (2023).

44. E. Galiffi, R. Tirole, S. X. Yin, H. N. Li, S. Vezzoli, P. A. Huidobro, M. G. Silveirinha, R. Sapienza, A. Alù, J. B. Pendry, Photonics of time-varying media. *Adv. Photonics* **4**, 014002 (2022).

45. Y. H. Tang, J. C. Fan, X. W. Li, J. Z. Ma, M. H. Qi, C. X. Yu, W. L. Gao, Physics-informed recurrent neural network for time dynamics in optical resonances. *Nat. Comput. Sci.* **2**, 169–178 (2022).

46. X. Chen, S. Xu, S. Shabani, Y. Zhao, M. Fu, A. J. Millis, M. M. Fogler, A. N. Pasupathy, M. Liu, D. N. Basov, Machine learning for optical scanning probe nanoscopy. *Adv. Mater.* 2109171 (2022).

47. B. K. P. Horn, B. G. Schunck, Determining optical flow. *Artif. Intell.* **17**, 185–203 (1981).

48. Y. Dai, Z. Zhou, A. Ghosh, R. S. K. Mong, A. Kubo, C. B. Huang, H. Petek, Plasmonic topological quasiparticle on the nanometre and femtosecond scales. *Nature* **588**, 616–619 (2020).

49. I. Amenabar, S. Poly, M. Goikoetxea, W. Nuansing, P. Lasch, R. Hillenbrand, Hyperspectral infrared nanoimaging of organic samples based on Fourier transform infrared nanospectroscopy. *Nat. Commun.* **8**, 14402 (2017).

50. M. Schnell, M. Goikoetxea, I. Amenabar, P. S. Carney, R. Hillenbrand, Rapid infrared spectroscopic nanoimaging with nano-FTIR holography. *ACS Photonics* **7**, 2878–2885 (2020).
